# The Structural and Electrophysiological Properties of Progesterone Receptor-Expressing Neurons Vary along the Anterior-Posterior Axis of the Ventromedial Hypothalamus and Undergo Local Changes across the Reproductive Cycle

**DOI:** 10.1523/ENEURO.0049-21.2021

**Published:** 2021-06-03

**Authors:** Inês C. Dias, Nicolas Gutierrez-Castellanos, Liliana Ferreira, Susana Q. Lima

**Affiliations:** Champalimaud Research, Champalimaud Centre for the Unknown, Av. Brasília s/n, 1400-038, Lisboa, Portugal

**Keywords:** ventrolateral part of the ventromedial hypothalamus, progesterone receptor, structural plasticity, whole-cell recordings, intrinsic excitability, reproductive cycle

## Abstract

Sex hormone levels continuously fluctuate across the reproductive cycle, changing the activity of neuronal circuits to coordinate female behavior and reproductive capacity. The ventrolateral division of the ventromedial hypothalamus (VMHvl) contains neurons expressing receptors for sex hormones and its function is intimately linked to female sexual receptivity. However, recent findings suggest that the VMHvl is functionally heterogeneous. Here, we used whole recordings and intracellular labeling to characterize the electrophysiological and morphologic properties of individual VMHvl neurons in naturally cycling females and report the existence of multiple electrophysiological phenotypes within the VMHvl. We found that the properties of progesterone receptor expressing (PR+) neurons, but not PR– neurons, depended systematically on the neuron’s location along the anterior-posterior (AP) axis of the VMHvl and the phase within the reproductive cycle. Prominent among this, the resting membrane potential of anterior PR+ neurons decreased during the receptive phase, while the excitability of medial PR+ neurons increased during the non-receptive phase. During the receptive phase of the cycle, posterior PR+ neurons simultaneously showed an increase in dendritic complexity and a decrease in spine density. These findings reveal an extensive diversity of local rules driving structural and physiological changes in response to fluctuating levels of sex hormones, supporting the anatomic and functional subdivision of the VMHvl and its possible role in the orchestration of different aspects of female socio-sexual behavior.

## Visual Abstract

**Figure:**
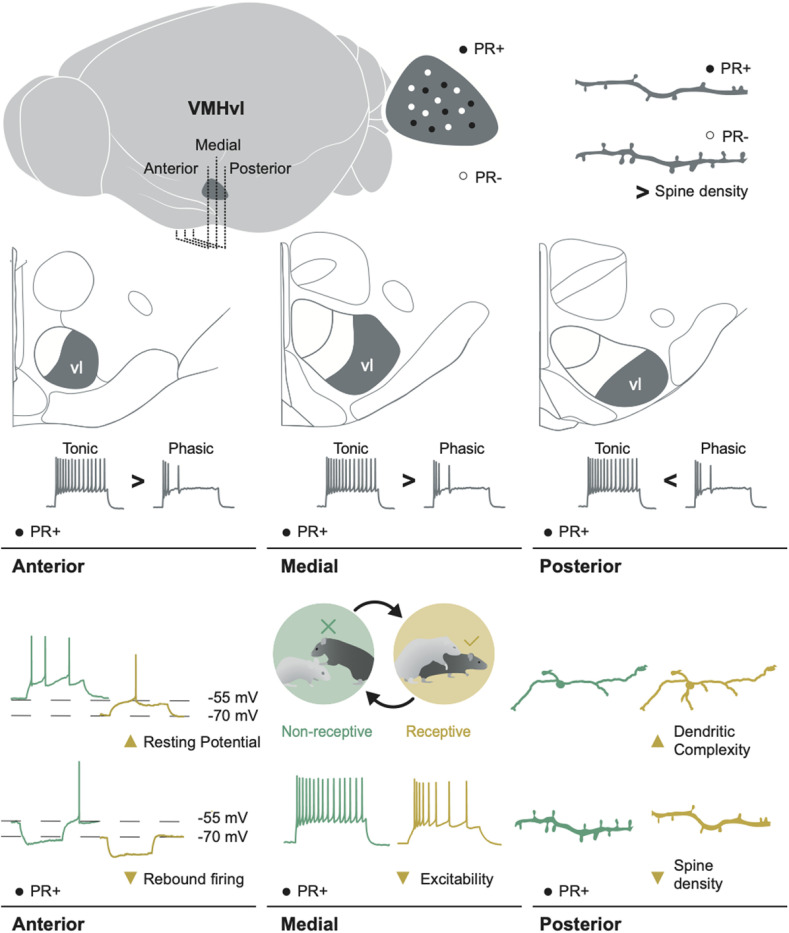

## Significance Statement

Fluctuating levels of sex hormones coordinate female behavior with their reproductive capacity by acting on neurons expressing their specific receptors. However, it is poorly understood how this seemingly simple message is integrated in local circuits to coordinate complex behavioral responses. Here, we show evidence that progesterone receptor expressing (PR+) neurons of the ventrolateral division of the ventromedial hypothalamus (VMHvl) undergo local and specific changes in structure and physiology, suggesting a compartmentalized action of the fluctuating systemic levels of sex hormone across the reproductive cycle, depending on their location in this hypothalamic nucleus.

## Introduction

Female reproductive physiology and behavior are under the control of the ovarian sex hormones estrogen and progesterone, whose fluctuating levels act reversibly in the female brain, organizing the activity of neural circuits to synchronize sexual behavior with reproductive capacity (Snoeren, 2018; Jennings and de Lecea, 2020). The ventrolateral division of the ventromedial hypothalamus (VMHvl) is crucial for female reproductive behavior, in particular for the display of lordosis, the female acceptance posture. Non-specific electrolytic lesions (Pfaff and Sakuma, 1979a) or ablation of genetically delineated neuronal populations (Rissman et al., 1997; Yang et al., 2013) of the VMHvl virtually abolish lordosis, while electrical stimulation at the same location enhances the probability of females displaying it (Pfaff and Sakuma, 1979b). Importantly, VMHvl neurons have rich expression of receptors for estrogen (ER) and progesterone (PR) and therefore are sensitive to the fluctuating levels of these sex hormones across the reproductive cycle (Snoeren, 2018; Jennings and de Lecea, 2020). In accordance, local infusion of sex hormones in the VMHvl increases female receptivity (Rubin and Barfield, 1983a), while male-evoked activity in this hypothalamic nucleus is enhanced when females are sexually receptive (Nomoto and Lima, 2015). Moreover, the output of PR-expressing neurons of the VMHvl is altered across the reproductive cycle and this cyclic remodeling is fundamental for the expression of acceptance when females are sexual receptive (Inoue et al., 2019). In summary, vast evidence highlights the importance of the VMHvl for coordinating female’s reproductive state with sexual receptivity.

Several studies have shown a large diversity of neuronal types with a wide variety of cellular identities within the VMHvl (McClellan et al., 2006; Flanagan-Cato, 2011; Kim et al., 2019). In addition, the generation of Cre lines providing access to ER-expressing and PR-expressing neurons uncovered further anatomic and functional subdivisions across the nucleus’ anterior-posterior axis (AP axis; McClellan et al., 2006; Flanagan-Cato, 2011; Hashikawa et al., 2017; Inoue et al., 2019; Kim et al., 2019; Lo et al., 2019; Wang et al., 2019), revealing a much broader role of this hypothalamic region in the control of different aspects of female socio-sexual behavior. While regulation of female sexual receptivity seems to be localized to the most posterior-lateral part of the VMHvl, its posterior-medial division is involved in aggressive behavior toward intruders in mothers (Hashikawa et al., 2017). Moreover, it was recently shown that ER-expressing neurons in the anterior portion of the VMHvl are important for self-defense in males (Wang et al., 2019). The connectivity of ER-expressing neurons also varies across the AP axis, further supporting the idea of topographic heterogeneity within this small nucleus (Lo et al., 2019).

How fluctuating levels of sex hormones affect the properties and function of VMHvl neurons remains poorly understood. Most studies aimed at elucidating the impact of sex hormones on neuronal activity in this region have focused on the structural properties of VMHvl neurons. The majority of neurons in the VMHvl have a long primary dendrite (LPD) that extends outside the nucleus, receiving inputs from other forebrain regions, a short primary dendrite (SPD), and secondary dendrites that may integrate local inputs (Millhouse, 1973; Calizo and Flanagan-Cato, 2000). Previous studies in rodents have shown that externally primed estradiol treatment shortens LPDs and reduces dendritic density in the VMHvl, effects which are reversed by progesterone (Griffin and Flanagan-Cato, 2008; Griffin et al., 2010). In addition, externally applied ovarian hormones increase the density of dendritic spines on the SPD of VMHvl neurons (Calizo and Flanagan-Cato, 2000), and decrease the spine density on the LPD of VMHvl neurons (Calizo and Flanagan-Cato, 2002). Moreover, treatment with estrogen was found to lead to an increase in the size of neuronal somata in the VMHvl (Carrer and Aoki, 1982; Griffin and Flanagan-Cato, 2011). More recently, it was shown that elevation in the concentration of circulating estrogen increases the number of axon terminals of PR-expressing VMHvl neurons that project to the anteroventral periventricular nucleus of the hypothalamus in female mice and that this plasticity is necessary for sexual receptivity (Inoue et al., 2019). Interestingly, aggressive behavior in males correlates with androgen levels in circulation and is dependent on the physiological and structural plasticity of ER-expressing neurons in the VMHvl (Stagkourakis et al., 2020). However, the electrophysiological properties of VMHvl neurons across the reproductive cycle have received very little attention (but see Kow and Pfaff, 1985; Booth et al., 2010).

A noteworthy common factor among the vast majority of previous studies on the effects of sex hormones on VMHvl neurons and behavior is that they have been performed in ovariectomized females in which sex hormones were systemically administered at concentrations that do not always match the physiological levels that are observed in naturally cycling females (Liu and Shi, 2015). Moreover, despite the growing evidence suggesting anatomic and functional diversity of the VMHvl, most studies have been largely focused on its most posterior levels (Yang et al., 2013; Hashikawa et al., 2017; Inoue et al., 2019). However, the VMHvl extends slightly more than a millimeter across the base of the hypothalamus (Kim et al., 2019; Lo et al., 2019), and sex hormone receptor expressing neurons are homogeneously distributed throughout (Sá and Fonseca, 2017; Kim et al., 2019). If and how the structural and physiological properties of VMHvl neurons along its AP axis vary across the female reproductive cycle remains elusive.

In order to gain a more complete understanding of the function of the VMHvl, in the present study we investigated the structural and electrophysiological properties of genetically delineated neurons along the VMHvl AP axis and across the reproductive cycle of naturally cycling females. We focused our efforts on neurons expressing the progesterone receptor (PR), as they are fundamental for female sexual behavior (Yang et al., 2013). To do so, we obtained slices from receptive and non-receptive females and performed in vitro whole-cell recordings of PR expressing (PR+) and PR non-expressing (PR–) neurons, with subsequent reconstruction of the recorded neurons to characterize their electrophysiological and structural properties.

Here, we report the existence of multiple electrophysiological phenotypes within the VMHvl and a wide variety of structural and physiological properties across the AP axis of the VMHvl, which are specific for PR+ neurons and that vary locally across the reproductive cycle. For instance, the membrane resting potential of anterior PR+ neurons decreases during the receptive phase, while the excitability of medial PR+ increases when females are non-receptive. During the receptive phase of the cycle, posterior PR+ neurons simultaneously undergo an increase in dendritic complexity and a decrease in spine density. These findings reveal an extensive diversity of local rules driving structural and physiological changes in response to fluctuating levels of sex hormones, supporting the anatomic and functional subdivision of the VMHvl and its possible role in the orchestration of different aspects of female socio-sexual behavior.

## Materials and Methods

### Animals

Data were collected from adult PR-Cre-R26R-EYFP (for short PR-EYFP) female mice (two to nine months). PR-EYFP mice express the enhanced yellow fluorescent protein (EYFP) in cells expressing PR (PR+) using the Cre-lox recombination system. Briefly, these mice result from the cross-breeding of B6129S(Cg)-Pgrtm1.1(Cre)Shah/AndJ75 mice (for short PRCre; JAX stock #017915; Yang et al., 2013), containing the Cre recombinase, which is expressed simultaneously with endogenous PR gene, with B6.129 × 1-Gt(ROSA26Sortm1(EYFP)Cos/J177 mice (for short R26R-EYFP; JAX stock #006148; Srinivas et al., 2001) that have the EYFP gene following a STOP sequence flanked by loxP. Therefore, in their offspring, heterozygous for both PR-Cre and R26R-EYFP alleles, loxP cassettes are removed by the action of Cre recombinase specifically in cells expressing PR, resulting in EYFP expression.

For the visualization of GABAergic somas, we used double heterozygous mice that resulted from the cross-breeding of B6J.129S6(FVB)-Slc32a1^tm2(cre)Lowl^/MwarJ (for short Vgat-ires-Cre knock-in; JAX stock #028862; Vong et al., 2011) with B6;129S6-Gt(ROSA)26Sor^tm9(CAG-tdTomato)Hze^/J (for short Ai9; JAX stock #007905; Madisen et al., 2010). Using the previously described Cre dependent recombinase method, these mice express the fluorescent marker tdTomato under the promoter of the vesicular transporter of GABA.

Animals were kept under controlled temperature of 23 ± 1°C and photoperiod of reversed 12/12 h light/dark cycle (light available from 8 P.M. to 8 A.M.) conditions and group-housed in standard cages with environmental enrichment elements. Food and water were provided *ad libitum*. Females were weaned at 20–21 d of age and group-housed with two to five animals.

After reaching six weeks of age, females were exposed to adult C57BL/6 male soiled bedding once per week to stimulate the natural reproductive cycle. All procedures were conducted in accordance with the animal protocols approved by the Portuguese National Authority for Animal Health (Direcção Geral de Alimentação e Veterinária; DGAV) and the Commission for Experimentation and Animal Welfare of the Champalimaud Centre for the Unknown (Órgão para o Bem Estar Animal; ORBEA).

### Reproductive cycle monitoring

To assess the reproductive state of naturally cycling female mice, vaginal cytology samples were collected every morning for at least one entire cycle (four/five consecutive days). This collection was done by vaginal lavage using a pipette (T-210-Y, AXYGEN, with its tip cut): 10 μl of 0.01 m PBS was gently flushed into the vagina and back out three or four times without touching the vaginal wall to avoid cervical stimulation and pseudopregnancy. The flush containing vaginal fluid was transferred to a glass slide and dried (Caligioni, 2009). Papanicolaou staining was used to differentiate cells (Bio-Optica protocol) and they were observed under a Zeiss AxioScope A1 brightfield microscope with a 10× objective. The identification of the reproductive cycle phase was done based on the proportion of each cell type in the smear. Proestrus is characterized by nucleated epithelial cells. In estrus the main cells present in the vaginal secretion are cornified squamous epithelial cells, clustered with irregular shape. These start to be replaced by leukocytes during metestrus, being these predominant cells in diestrus (Pfaus, 1999; Caligioni, 2009; Snoeren, 2018). Experiments were performed when females were in proestrus/estrus (sexually receptive) or in diestrus (sexually non-receptive).

### *Ex vivo* electrophysiological recordings

PR-Cre-EYFP mice were deeply anesthetized with isoflurane and decapitated 1–2 h after the phase of the cycle identification by pap smearing. This protocol is used by the laboratory for behavioral experiments (Nomoto and Lima, 2015). After decapitation, brains were quickly removed and placed into “ice-cold” slicing solution containing the following: 0.66 mm kynurenic acid, 3.63 mm pyruvate, 2.5 mm KCl, 1.25 mm NaH_2_PO_4_, 26 mm NaHCO_3_, 10 mm D-glucose, 230 mm sucrose, 0.5 mm CaCl_2_, 10 mm MgSO_4_, and bubbled with 5% CO_2_ and 95% O_2_. Coronal sections with 300 μm containing the VMH were cut using a vibratome (Leica VT1200) in the same ice-cold slicing solution. Slices were recovered in oxygenated artificial CSF (ACSF) containing the following: 127 mm NaCl, 2.5 mm KCl, 25 mm NaHCO_3_, 1.25 mm NaH_2_PO_4_, 25 mm D-glucose, 2 mm CaCl_2_, and 1 mm MgCl_2_, at 34°C for 30 min and stored in the same solution at room temperature before being transferred to the recording chamber.

Whole-cell recordings were made under a SliceScope Pro (Scientifica) microscope with the slices submerged in ACSF. A beam of blue light generated with a LED illumination system (CoolLED, pE300) was passed through an immersion objective (Olympus, 40×) to achieve wide field illumination of the slice. PR+ neurons were identified by their natural fluorescence under the microscope and PR– by the absence of fluorescence. A standard GFP filter cube (Thorlabs) was used to filter EYFP emission. EYFP expression, and therefore fluorescence levels, are independent of the reproductive cycle, as after Cre recombination, the expression of the fluorescent protein is dependent on the rosa locus. Patch recording pipettes (resistance 3–5 MΩ) were filled with internal solution containing the following: 135 mm K-gluconate, 10 mm HEPES, 10 mm Na-phosphocreatine, 3 mm Na-L-ascorbate, 4 mm MgCl_2_, 4 mm Na-ATP, 0.4 mm Na-GTP (pH 7.2 adjusted with NaOH and osmolarity ∼292 mOsm), and 0.1% of biocytin. Internal solution was filtered with a 0.2-μm pore size cellulose acetate filter tip. A Multiclamp 700B amplifier and digitized at 10 kHz with a Digidata 1440a digitizer (both from Molecular Devices) were used and the data were filtered online with a 10-kHz low-pass filter.

We applied a test-pulse of –10 mV for 100 ms in voltage-clamp mode at –70 mV to monitor membrane capacitance (Cmem) and series resistance. To determine excitability and firing properties, current-clamp experiments were performed at resting potential by applying series of 1000-ms current pulses between 0 and 240 pA with an increasing interval of 30 pA at a frequency of 0.1 Hz. Hyperpolarization-activated inward current (Ih current) and rebound firing were measured with a 1000-ms current pulse at –90 pA. The bridge balance was semi-automatically compensated using Axon pCLAMP 10 (Molecular Devices).

After recording, the pipette was carefully withdrawn from the neurons to allow the resealing of the membrane and the diffusion of biocytin inside the neuron. Slices were then transferred to a multi-well plate containing 4% paraformaldehyde (PFA) for ∼1 h for fixation and then transferred to 0.01 m PBS for storage before immunostaining.

### Histology

To visualize biocytin filling, slices were incubated in streptavidin-Alexa Fluor 488 conjugate (Invitrogen; concentration, 1:200) with 0.3% Triton X-100 in 0.01 m PBS at room temperature for 3 h in dark. After washing three times with 0.01 m PBS for 30 min each, sections were mounted in glass slides (Menzel–Glazer), coverslipped (Marienfeld) with Mowiol mounting medium (Sigma-Aldrich) and the edges sealed with clear nail polish. For tissue collection, VGat-Cre-tdtomato mice were deeply anesthetized with a lethal amount of a mixture of 12% of ketamine (Imalgene 1000, Merial) and 8% of xylazine (Rompun 2%, Bayer) in saline solution and perfused transcardially with 0.01 m PBS followed by 4% PFA in PBS. The brains were removed, fixated in the 4% PFA solution for ∼1 h and transferred to a 30% sucrose (Sigma-Aldrich) in 0.01 m phosphate buffer and 0.1% sodium azide (ACROS Organics) to allow cryopreservation. Brains were then frozen and coronal sections with 45-μm thickness were obtained from the VMH using a freezing sliding microtome (SM2000R, Leica).

Histologic sections and fixed *ex vivo* brain slices were imaged with a Zeiss LSM 710 confocal laser scanning microscope with a 10× and a 25× magnification objectives.

### Electrophysiological data analysis

**Figure 1.**
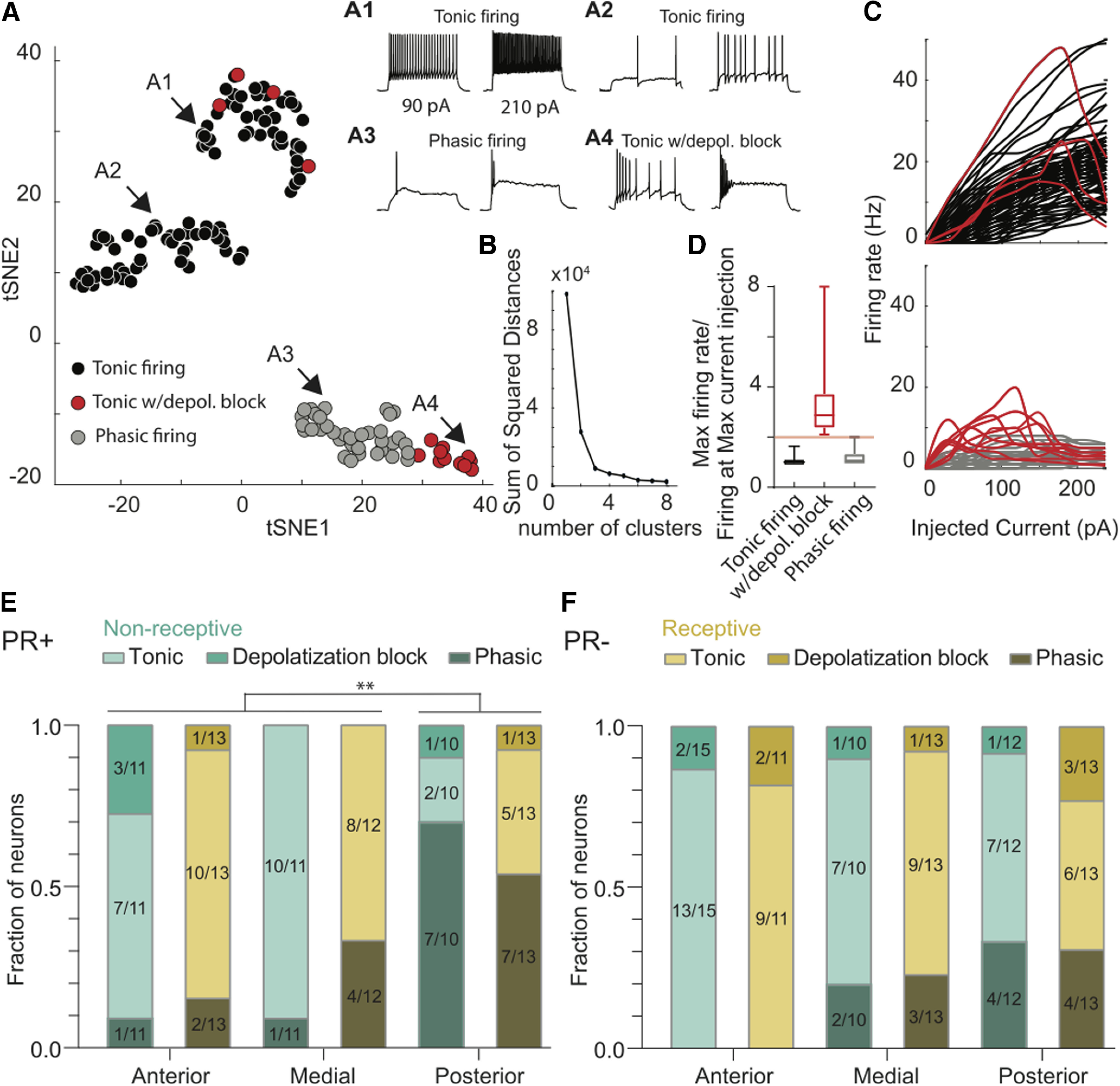
The predominant firing pattern of PR+ neurons varies across the AP axis and differs from the PR– population. ***A***, Low-dimensional embedding of VMHvl neurons input current to output voltage responses (I-V curves) using tSNE. Four representative examples of the different firing responses found correspond to tonic neurons with high firing rate (***A1***), tonic neurons with low firing rate (***A2***), phasic neurons (***A3***), and tonic neurons with depolarization block (***A4***). ***B***, Sum of squared distances between each data point and its assigned cluster as a function of increasing number of possible clusters. ***C***, ***D***, Tonic neurons with depolarization block, which initially were largely clustered with the phasic neurons, can be distinguished by obtaining the ratio between the maximum firing rate and the firing rate at the maximum current injected (240 pA). ***E***, The majority of PR+ neurons in the anterior and medial VMHvl display tonic firing, while the posterior PR+ neurons have mainly phasic firing (*p* < 0.001), with no changes across the reproductive cycle. ***F***, The PR– neurons display mainly tonic firing across the whole AP axis and do not change across the reproductive cycle. χ^2^ test; **p < 0.01

A total of 144 recorded neurons were used in the present study. Please see Table 1 and Figure 1 for the numbers of recorded PR+ and PR– neurons, across the reproductive cycle and AP axis.

**Table 1:** Passive membrane properties of the PR+ and PR– populations across the reproductive cycle and the AP axis

	PR+ neurons											
	Non-receptive						Receptive					
	Anterior (*n* = 10)		Medial (*n* = 8)		Posterior (*n* = 11)		Anterior (*n* = 9)		Medial (*n* = 11)		Posterior (*n* = 11)	
	T	P	T	P	T	P	T	P	T	P	T	P
Capacitance (pF)	9.03 (2.31)		11.57 (2.66) L1*		7.34 (5.02) L1*		11.45 (3.04)		10.04 (2.256) L1*		9.57 (2.70) L1*	
	9.08	8.99	11.59	7.83	8.21	7.34	11.45	9.87	10.04	9.26	9.57	10.17
Membrane resistance (MΩ)	970.64 (1012.68)		788.77 (384.09)		912.00 (1209.69)		998.98 (604.43)		823.46 (853.00)		1082.56 (687.92)	
	1017.11	570.19	744.54	1231.50	999.65	912.00	998.98	1326.90	724.09	1116.09	995.93	1082.56
Time constant (ms)	0.31 (0.09) L2*		0.36 (0.16)		0.22 (0.08) L2*		0.36 (0.18) L2*		0.27 (0.20)		0.24 (0.16) L2*	
	0.32	0.28	0.36	0.18	0.24	0.20	0.34	0.42	0.26	0.27	0.30	0.23
	PR– neurons											
	Non-receptive						Receptive					
	Anterior (*n* = 16)		Medial (*n* = 10)		Posterior (*n* = 10)		Anterior (*n* = 11)		Medial (*n* = 11)		Posterior (*n* = 13)	
	T	P	T	P	T	P	T	P	T	P	T	P
Capacitance (pF)	11.04 (4.70)		10.05 (1.89) L1*		8.82 (2.53) L1*		10.54 (2.90)		9.54 (1.28) L1*		10.53 (2.20) L1*	
	11.04	-	10.54	8.69	8.82	8.71	10.54	-	9.53	10.02	10.16	11.60
Membrane resistance (MΩ)	808.55 (438.933)		1025.83 (425.53)		861.21 (284.09)		1258.25 (1412.35)		884.96 (654.043)		871.47 (556.16)	
	808.55	-	1142.86	781.92	873.94	861.21	1258.25	-	888.20	884.96	830.55	1160.18
Time constant (ms)	0.36 (0.19) L2*		0.34 (0.18)		0.35 (0.17) L2*		0.36 (0.08) L2*		0.30 (0.11)		0.28 (0.16) L2*	
	0.36	-	0.38	0.23	0.34	0.37	0.36	-	0.29	0.34	0.28	0.32

T, tonic neurons; P, phasic neurons. Values expressed as median.

Capacitance: L1*, location/phase interaction *F*_(2,132)_ = 3.20, *p* = 0.04; *τ*: L2*, location *F*_(2,132)_ = 3.23, *p* = 0.04. Three-way ANOVA, Sidak correction for multiple comparison. Values expressed as median (IQR); *p < 0.05.

The action potentials recorded in current-clamp mode were analyzed with custom-written MATLAB code. Each spike within the spike trains obtained on current injection were detected and used to determine the firing frequency, interspike interval (ISI), threshold to spike, latency to spike and coefficient of variation calculated at a fixed current injection of 150 pA). The coefficient of variation was calculated using the following formula (Holt et al., 1996):

$$CV2=\frac{2\left|ISI_{n+1}-ISI_{n}\right|}{\left(ISI_{n+1}+ISI_{n}\right)}.$$

The rebound action potentials observed after injecting hyperpolarizing currents were obtained using the same method.

The first action potential obtained of each spiking sweep was used to calculate the spike amplitude and spike half-width to prevent frequency-dependent artifacts in the spike shape caused by sodium and potassium channel inactivation.

The resting potential was determined by averaging the membrane voltage values before the current injection and the Ih current amplitude was calculated by the difference between the early and late phase of a hyperpolarizing current pulse.

Voltage clamp test-pulses were analyzed using Clampfit 10.7 Software (Molecular Devices, LLC) and access resistance (Ra), membrane resistance (Rm), time constant (*τ*), and capacitance were calculated. Only neurons with Ra 1/10th lower than the Rm were used for analysis. Ra was comparable across location, genotype and the phase of the cycle in the present study.

The passive membrane properties Rm, Cmem, and membrane *τ* were obtained immediately after membrane rupture, using a square voltage step (−10 mV, 100 ms). The Ra was determined by measuring the amplitude of the current response to the command voltage step and the Rm as the difference between the baseline and the holding current in the steady state after the capacitive decay, by applying the Ohm’s law. The membrane *τ* was determined by a single exponential fit of the decay phase in response to the square pulse. An approximation of the capacitance was made by using the formula:

τ=membranecapacitance * accessresistance.

The principal component analysis (PCA)-initialized t-distributed stochastic neighbor embedding (tSNE) on the firing rates and its subsequent clustering were performed using the *tsne* and *kmeans* functions of MATLAB 2020a.

### Neuronal reconstruction and image data analysis

A total of 180 neurons VMHvl neurons were filled with biocytin in acute slices obtained from PR-EYFP female mice using whole-cell recording pipettes. These slices containing biocytin filled neurons were fixed and stained with streptavidin-Alexa Fluor 488 conjugated for the subsequent reconstruction of neuronal structure. In our hands, the native EYFP signal systematically bleached after slice fixation, being several fold dimmer than the fluorescence obtained with streptavidin-Alexa Fluor 488. Therefore, with our confocal settings, no somatic EYFP signal was obtained despite their spectral overlap. Please refer to Table 2 for the numbers of filled PR+ and PR– neurons across the reproductive cycle and AP axis.

**Table 2.** Morphologic profile of PR+ and PR– neurons across the reproductive cycle and the AP axis

	PR+ neurons					
	Non-receptive			Receptive		
	Anterior (*n* = 19)	Medial (*n* = 17)	Posterior (*n* = 20)	Anterior (*n* = 15)	Medial (*n* = 19)	Posterior (*n* = 16)
Soma area (μm^2^)	184.88 (68.97)	217.01 (95.72)	193.08 (53.31)	219.77 (75.28)	182.92 (38.98)	219.31 (53.94)
Dendrites/neuron	7.00 (2.00)	7.00 (3.00)	6.00 (3.50)	7.00 (3.00)	9.00 (3.00)	7.50 (1.00)
Primary dendrites/neuron	4.000(1.00) L1*	4.000(1.00) L1*	3.50 (1.00)	4.000(1.00) L1*	4.000(1.00) L1*	3.50 (1.75)
Branch points/neuron	3.00 (2.00)	3.00 (3.00)	3.00 (3.00)	4.00 (3.00)	5.00 (4.00)	4.00 (1.75)
Dendritic length of LPDs	572.69 L2* (199.05)	448.43 L2* (268.22)	337.53 L2* (287.65)	640.69 L2* (277.72)	473.32 L2* (273.33)	482.57 L2* (202.10)
Average dendritic length of SPDs	205.36 (102.82)	214.50 (65.76)	145.53 (93.24)	181.54 (71.97)	170.55 (77.82)	177.85 (75.57)
	PR– neurons					
	Non-receptive			Receptive		
	Anterior (*n* = 16)	Medial (*n* = 12)	Posterior (*n* = 15)	Anterior (*n* = 10)	Medial (*n* = 12)	Posterior (*n* = 14)
Soma area (μm^2^)	183.64 (58.52)	208.24 (66.82)	171.12 (105.02)	177.00 (124.49)	188.30 (112.71)	184.28 (68.46)
Dendrites/neuron	8.00 (4.00)	7.50 (3.50)	7.00 (3.00)	7.00 (1.50)	7.00 (2.50)	7.00 (2.75)
Primary dendrites/neuron	4.000(1.75) L1*	3.000(1.00) L1*	4.00 (2.00)	3.500(2.25) L1*	3.000(0.75) L1*	4.00 (1.25)
Branch points/neuron	4.00 (5.50)	4.00 (3.50)	3.00 (2.00)	3.00 (3.25)	4.00 (1.00)	3.00 (3.25)
Dendritic length of LPDs	642.64 L2* (235.57)	644.05 L2* (96.20)	509.16 L2* (253.81)	566.10 L2* (352.88)	494.52 L2* (289.21)	389.96 L2* (194.99)
Average dendritic length of SPDs	155.87 (62.25)	255.72 (102.05)	172.95 (79.60)	124.89 (60.77)	150.58 (134.84)	176.90 (85.55)

Dendrites per neuron: genotype/phase interaction *F*_(1,173)_ = 7.85, *p* = 0.01; branch points per neuron: genotype/phase interaction *F*_(1,173)_ = 8.81, *p* = 0.02. L1*, primary dendrites per neuron: location *F*_(2,173)_ = 3.14, *p* = 0.04. L2*, length of LPDs: genotype/phase interaction *F*_(1,168)_ = 6.67, *p* = 0.01, location *F*_(2,168)_ = 9.73, *p* < 0.0001. Three-way ANOVA, Sidak correction for multiple comparison. Values expressed as median (IQR); *p < 0.05

To determine the bregma coordinate for each reconstructed neuron and ensure that its location fell within the VMHvl, in a separate experiment we first assessed the extension of the VMHvl, taking advantage of the VGAT-tdTomato. The VMH is organized in a core of glutamatergic neurons (Ziegler et al., 2002; Yamamoto et al., 2018) and a surrounding shell with higher density of GABAergic neurons (Yamamoto et al., 2018). Therefore, we assessed the AP extension of the VMH by counting consecutive slices in which the VMH was detectable, as shown by a decrease of the VGAT-tdTomato signal (Fig. 6*A*) and multiplying by the thickness of the histologic slices. A sharp transition from low to high tdTomato fluorescence was observed both in its anterior limit (near the anterior hypothalamus) as well as with its posterior limit (near the premammilary hypothalamus; data not shown). The extension obtained matched the extension illustrated by the Paxinos Brain Atlas: anterior limit = 1.05 to posterior limit = 2.06 from bregma (that is, the VMHvl spans for ∼1 mm). For each mouse, we systematically sampled three consecutive levels of the VMH in slices of 300 μm of thickness. We thus categorized each sampling level as anterior (bregma −1.05 to −1.30 mm approximately), medial (bregma −1.30 to −1.65 mm approximately), and posterior (bregma −1.65 to −2.00 mm approximately) levels of the VMH (Fig. 6*B*,*C*). Localization of the reconstructed neurons in the brain was assessed by matching in Adobe Illustrator CS6 (Adobe Systems Incorporated) the confocal images of the neurons with adapted sections from the Paxinos brain atlas (Franklin and Paxinos, 2008). Only the neurons inside the defined boundaries of the VMHvl were considered for quantification. We used the simple neurite tracer (Longair et al., 2011) package from Fiji/ImageJ software (Schindelin et al., 2012) to analyze the morphologic properties and Sholl profiles of the neurons filled with biocytin. The Cell Counter Fiji/ImageJ plugin was used to manually count spines.

Cut and resealed dendrites (identified as a globular thickening in the extreme of a dendrite) were quantified for every neuron and did not reveal significant differences across groups (Number of cut dendrites/total number of dendrites quantified per group for PR+ neurons: 21/126 in anteriorNon-Rec, 19/120 in anteriorRec, 20/116 in medialNon-Rec, 31/151 in medialRec, 26/131 in posteriorNon-Rec and 26/121 in posteriorRec. And for PR– neurons: 26/130 in anteriorNon-Rec, 16/70 in anteriorRec, 19/95 in medialNon-Rec, 15/85 in medialRec, 26/100 in posteriorNon-Rec and 17/100 in posteriorRec). When tested with a Fisher’s exact test, neither PR+ nor PR– revealed statistical difference in the proportion of cut dendrites. We thus assume that this unavoidable artifact of our method of neuronal recording and reconstruction did not add any group-specific bias in the structural quantification.

In our hands, as well as in previous studies (Calizo and Flanagan-Cato, 2000), the intracellular labeling of VMHvl neurons did not always make their thin axonal process visible, and therefore, the axons identified were not considered for analysis.

Graphs were made using custom made MATLAB code or GraphPad Prism 8 Software.

### Statistical analyses

Statistical analyses were performed using GraphPad Prism 8 Software. Normality of the residuals was tested with the D’Agostino–Pearson omnibus K2 test. When normally distributed, three-way ANOVA tests were performed to compare groups in different phases of the cycle (D vs PE) versus location in the AP axis (anterior vs medial vs posterior) versus genotype (PR+ and PR– neurons), using the Sidak test to correct for multiple comparisons. In Figure 5*E*, a two-way ANOVA, with Sidak test for multiple comparisons, was used to investigate the origin of the differences in the location factor that were specific for PR– neurons. In the properties whose residuals did not pass the normality test, a logarithmic or square root transformation was applied and normality was consequently reassessed. As specified in the legend, in the properties that the transformations did not make the residuals become normally distributed, we performed a Kruskal–Wallis test followed by a Dunn’s *post hoc* test for multiple comparisons.

**Figure 2.**
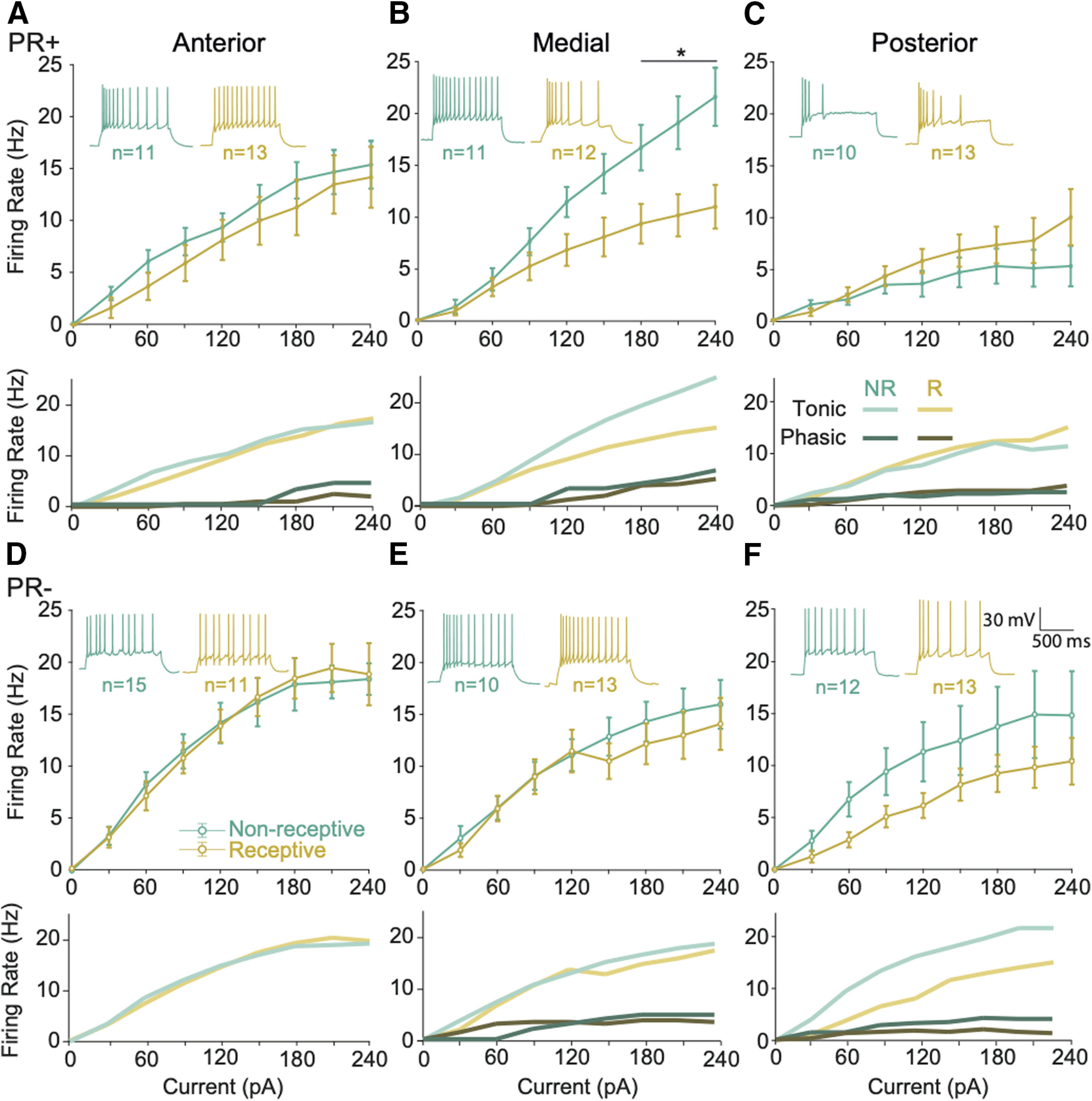
The excitability of medial PR+ neurons changes across the reproductive cycle. ***A–C***, top, Group mean and SEM of I-V curves across the reproductive cycle reporting the firing frequency of PR+ neurons after injecting 1000-ms current pulses between 0 and 240 pA (30-pA steps), reporting changes only in (***B***) medial PR+ neurons (*F*_(8,160)_ = 6.87, *p* < 0.0001, multiple comparison *p* = 0.02 at 180 pA, *p* < 0.01 at 210 pA, *p* < 0.001 at 240 pA). Bottom, Mean of tonic and phasic firing responses per group. Insets, Representative recordings of voltage response from PR+ neurons in response to the same current injection. Anterior *n* = 11 NR, *n* = 13 R, medial *n* = 11 NR, *n* = 12 R, posterior *n* = 10 NR, *n* = 13 R. ***D–F***, IV curves across the reproductive cycle reporting no changes in the firing frequency of PR– neurons after injecting 1000-ms current pulses between 0 and 240 pA (30-pA steps). Insets, Representative recordings of voltage response from PR– neurons in response to the same current injection. Anterior *n* = 15 NR, *n* = 11 R, medial *n* = 10 NR, *n* = 13 R, posterior *n* = 12 NR, *n* = 13 R. ***A***, ***C***, ***D***, ***F***, PR+ neurons are less excitable than PR– in the anterior and posterior VMHvl (*F*_(8,360)_ = 3.53, *p* < 0.001 and *F*_(8,361)_ = 2.83, *p* < 0.01, respectively) but not in (***B***, ***E***) the medial. Mixed-effects test with repeated measures. Mean (±SEM); *p < 0.05

For Figure 2*A–F*, a mixed-effects test with repeated measures, and Figure 7*C–H* a two-way ANOVA with repeated measures were performed. For Figures 1*E–F*, 5*B*,*C*, a χ^2^ test was performed, the pairwise comparisons we tested followed the same structure as the one obtained for the three-way ANOVA multiple comparisons. Whisker plots represent median with interquartile range. Error bars represent mean ± SEM *p* values were rounded to two decimal places. Significance was noted as *p < 0.05, **p < 0.01.

## Results

### The proportion of tonic and phasic firing PR+ neurons varies across the anteroposterior axis

In order to investigate the neuronal excitability of PR+ and PR– neurons, we recorded current to voltage input-output response curves (I-V curves) at resting potential in current-clamp mode in acute slices of naturally cycling PR-Cre x EYFP adult female mice. Briefly, the female reproductive stage was determined by vaginal lavage and slices were obtained from females in the least receptive state (diestrus) and the most receptive state (proestrus/estrus). PR+ neurons were identified by their natural fluorescence under the microscope and PR– by the absence of fluorescence (for details, see Materials and Methods).

We performed a PCA-initialized tSNE analysis on the I-V curve firing rates obtained for our full sample of recorded PR+ and PR– neurons. To understand how many clusters can be optimally distinguished in the resulting embedded data (Fig. 1*A*), we performed k-means clustering with a possible number of clusters varying between one and eight. When quantifying the sum of squared distances between each datapoint and its assigned cluster centroid over an increasing number of clusters, we observed a characteristic “elbow” shape when there were three possible clusters, suggesting this is the optimal number of clusters in our dataset (Fig. 1*B*). The first two clusters corresponded to tonic neurons with high and low firing rates (Fig. 1*A1*,*A2*, respectively). Tonic firing neurons exhibited a linear increase in firing rate in response to the increases in the magnitude of current injected (Fig. 1*C*, top). The third cluster roughly corresponded to phasic firing neurons (Fig. 1*A3*), neurons in which the action potentials did not linearly increase in response to increased current injection (Fig. 1*C*, bottom). These neurons exhibited a step function-like behavior, where after reaching the firing threshold, the number of action potentials remained constant with increased amounts of current. However, when visually inspecting the firing profiles of neurons in this cluster (Fig. 1*C*), we observed that this cluster included phasic neurons and neurons in which the firing rate initially increased linearly but then entered depolarization block and drastically reduced their firing rate (Fig. 1*A4*). When dividing the peak firing rate of each neuron by their firing rate at the maximum current injected (240 pA), a subset of neurons with particularly high ratio values was clearly distinguishable from the rest (Fig. 1*D*). These neurons were tonic firing neurons with depolarization block.

We therefore considered two major categories of neurons. The first category being tonic firing neurons, comprising neurons that did not reach the depolarization block within the range of currents we injected (Fig. 1*A1*,*A2*, tonic neurons), and neurons that did (Fig. 1*A4*, tonic neurons with depolarization block), independently of their firing rate. And the second category being phasic firing neurons (Fig. 1*A3*).

The majority of the anterior and medial PR+ neurons displayed tonic firing, while most of the posterior ones displayed phasic firing (χ^2^ test, *p* < 0.001; Fig. 1*E*), with no differences across the reproductive cycle. In contrast, the PR– population is homogenously composed of a higher proportion of tonic firing neurons across the AP axis (Fig. 1*F*).

To summarize, the VMHvl is composed of multiple electrophysiological phenotypes and posterior PR+ neurons have a higher density of phasic neurons in comparison to the anterior and medial levels of the VMHvl.

### Local changes in the intrinsic excitability and threshold to spike of PR+ neurons across the reproductive cycle

The analysis of the I-V curves revealed that in the anterior and posterior VMHvl, the excitability of PR+ neurons does not to change across the cycle (Fig. 2*A*,*C*). In contrast, medial PR+ neurons of non-receptive females showed significantly higher excitability when compared with the excitability of medial PR+ neurons originating from receptive females, particularly in response to higher input current (Fig. 2*B*, top, strong interaction between the phase of the cycle and the amount of current injected; mixed-effects test, *F*_(8,160)_ = 6.87, *p* < 0.0001). This modulation is caused by a change in the excitability of the tonic firing medial neurons across the reproductive cycle while the behavior of medial phasic neurons remained unchanged (Fig. 2*B*, bottom). In addition, this change was specific to the PR+ population since no changes were observed across the cycle in the PR– neurons at any of the AP levels (Fig. 2*D–F*).

We also observed that the PR+ population was less excitable than the PR– in the anterior and posterior levels of the VMHvl (mixed-effects test, anterior PR+ vs anterior PR– *F*_(8,360)_ = 3.53, *p* < 0.001 and posterior PR+ vs posterior PR– *F*_(8,361)_ = 2.83, *p* < 0.01) but not in the medial subdivision, probably in part because of the increased excitability of medial PR+ neurons from non-receptive females.

It is worth mentioning that the Rm did not vary across experimental groups (Table 1) and only a small decrease was observed in the capacitance of the anterior and posterior PR+ of non-receptive females (three-way ANOVA, location/phase interaction *F*_(2,132)_ = 3.20, *p* = 0.04). The membrane *τ* also did not vary across the reproductive cycle, however posterior PR+ neurons exhibited a smaller *τ* when compared with the anterior PR+ population (three-way ANOVA, location *F*_(2,132)_ = 3.23, *p* = 0.04, multiple comparison anterior vs posterior *p* = 0.04).

**Figure 3.**
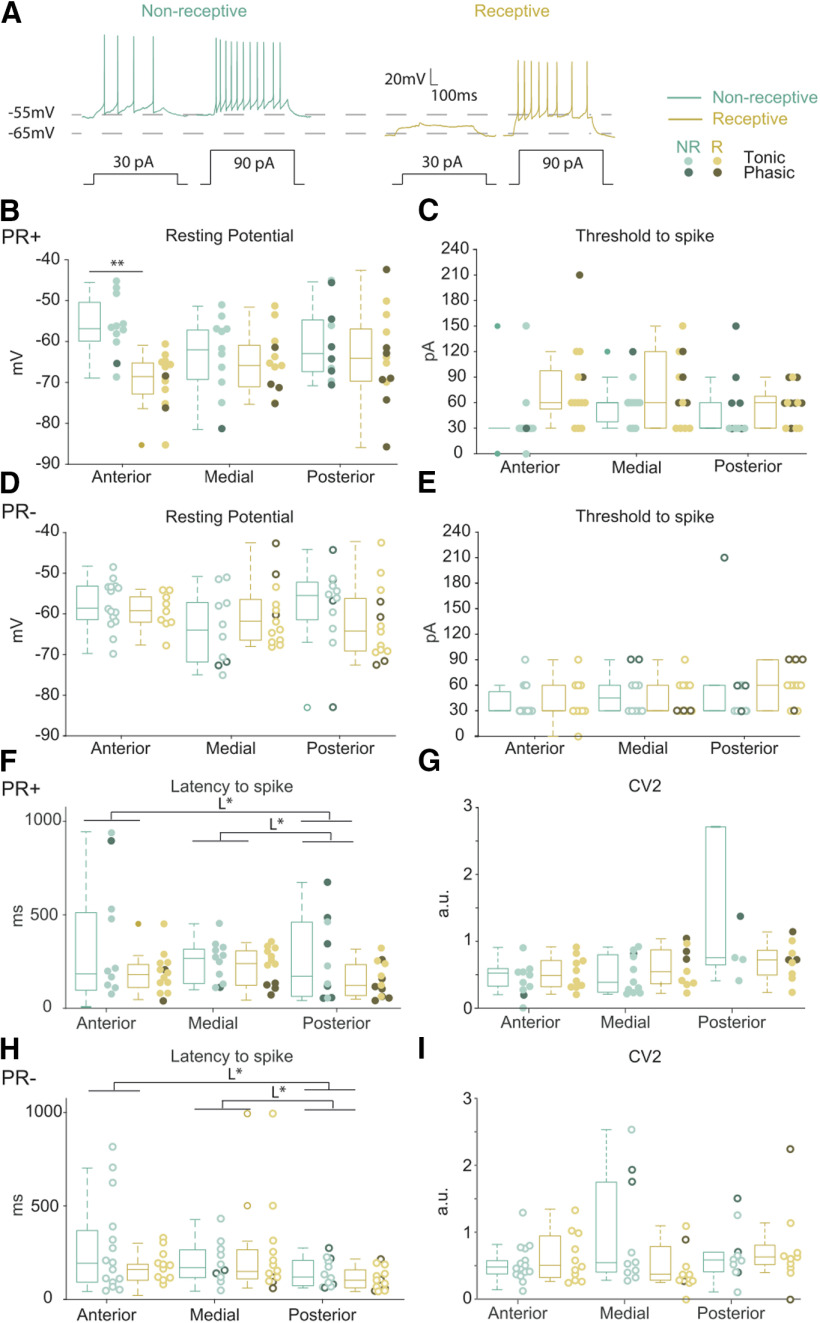
The resting membrane potential of anterior PR+ neurons changes across the reproductive cycle. ***A***, Representative membrane voltage recordings from PR+ neurons in response to 30 and 90 pA. ***B***, Anterior PR+ neurons of receptive females have a lower resting potential compared with non-receptive females (phase *F*_(1,130)_ = 6.39, *p* = 0.01, multiple comparison antNon-Rec+ vs antRec+ *p* < 0.01). The threshold to spike (***C***), latency to spike (***F***), and regularity of the spike train (CV2; ***G***) of the PR+ neurons do not change. Anterior *n* = 11 NR, *n* = 13 R, medial *n* = 11 NR, *n* = 12 R, posterior *n* = 10 NR, *n* = 13 R. Resting potential (***D***), threshold to spike (***E***), latency to spike (***H***), and regularity of the spike train (CV2; ***I***) do not vary in PR– neurons across the reproductive cycle. Anterior *n* = 15 NR, *n* = 11 R, medial *n* = 10 NR, *n* = 13 R, posterior *n* = 12 NR, *n* = 13 R. ***F***, ***H***, There is a mild lower latency to spike of the posterior neurons independently of the cycle and genotype (location *F*_(2,132)_ = 5.48, *p* = 0.01, L*, multiple comparison ant vs posterior *p* = 0.01 and medial vs posterior *p* = 0.01). ***B***, ***D***, ***F***, ***H***, Three-way ANOVA, Sidak correction for multiple comparison. ***C***, ***E***, ***G***, ***I***, Kruskal–Wallis test, with multiple comparisons. Box plots represent medians (±25th–75th percentile); *p < 0.05, **p < 0.01. Individual data points are shown per group color coded according their tonic or phasic firing type.

The resting potential of anterior PR+ neurons of receptive females was lower when compared with non-receptive females (Fig. 3*A*,*B*; three-way ANOVA, phase *F*_(1,132)_ = 6.39, *p* = 0.01, multiple comparison antNon-Rec vs antRec *p* < 0.01). However, this difference was not large enough to produce changes in the threshold to spike (Fig. 3*C*). The latency to spike of PR+ and PR– neurons did not vary across the cycle (Fig. 3*F*,*H*). In addition, we observed a mild, yet significant, lower latency to spike of the posterior neurons when compared with anterior (Fig. 3*F*,*H*; three-way ANOVA, location *F*_(2,132)_ = 5.48, *p* < 0.01, multiple comparison anterior vs posterior *p* = 0.01) and medial neurons (multiple comparison medial vs posterior *p* = 0.01) that was independent of the phase of the cycle and the genotype.

The regularity of the recorded spike trains was similar across experimental groups, as shown by comparable CV2 values across phase and location for both the PR+ and PR– populations (regularity was determined at 150 pA of injected current; Fig. 3*G*,*I*).

**Figure 4.**
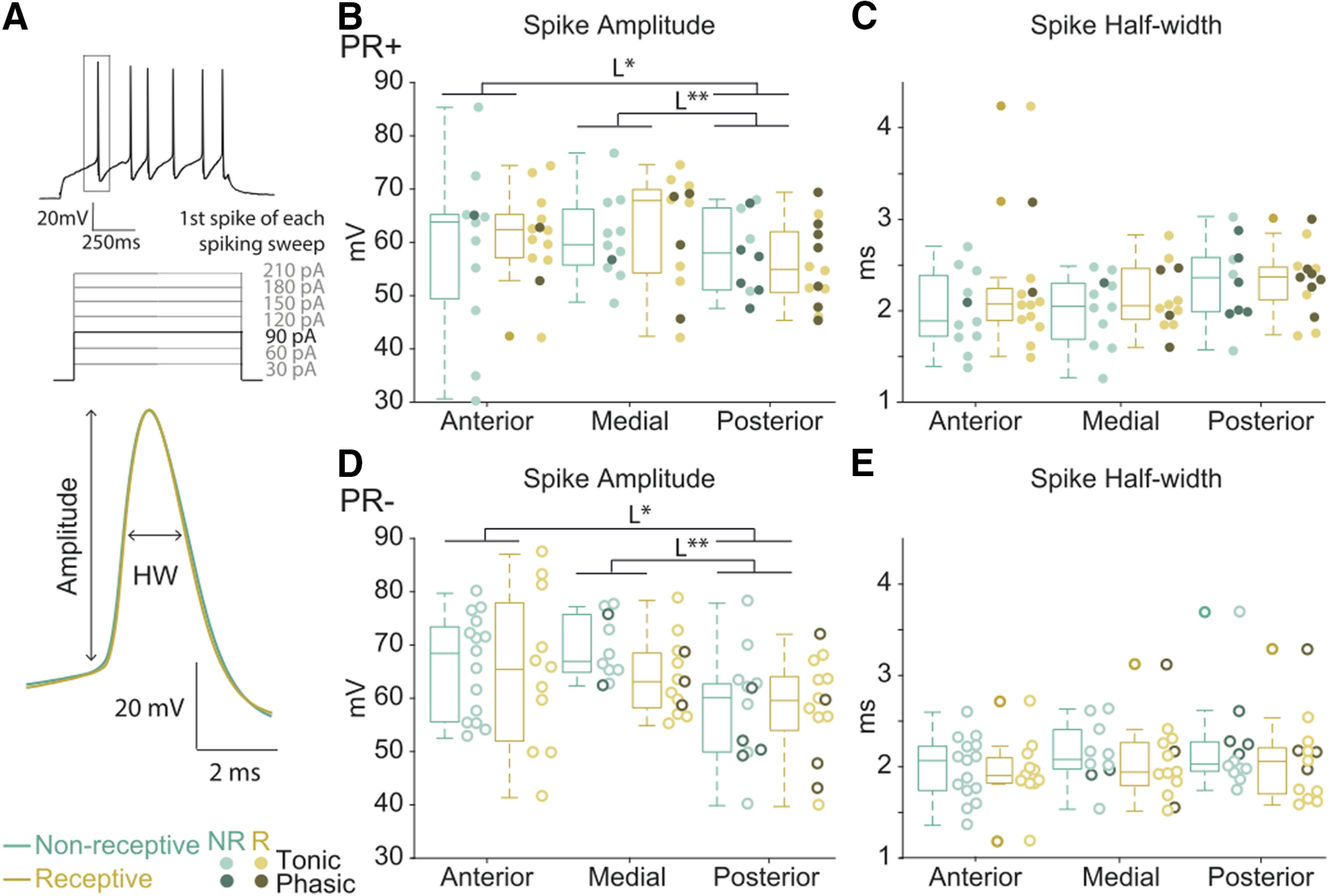
The spike waveform of PR+ and PR– neurons is consistent across the reproductive cycle. ***A***, Illustration of the recording protocol used to quantify action potential properties and representative examples of the average spike waveform illustrating the amplitude and the half-with (HW). ***B***, ***D***, The amplitude of the first action potential for each current injection step of the PR+ and PR– neurons does not change across the reproductive cycle; however, it differs mildly across the AP axis (location *F*_(2,132)_ = 5.74, *p* < 0.01, multiple comparison L*, anterior vs posterior *p* = 0.03 and L**, medial vs posterior *p* < 0.01). ***C***, ***E***, The spike half-width of PR+ and PR– neurons is consistent across the reproductive cycle and the AP axis. PR+ anterior *n* = 11 NR, *n* = 13 R, medial *n* = 11 NR, *n* = 12 R, posterior *n* = 10 NR, *n* = 13 R. PR– anterior *n* = 15 NR, *n* = 11 R, medial *n* = 10 NR, *n* = 13 R, posterior *n* = 12 NR, *n* = 13 R. Three-way ANOVA, Sidak correction for multiple comparison. Medians (±25th–75th percentile); *p < 0.05, **p < 0.01. Individual data points are shown per group color coded according their tonic or phasic firing type.

In focusing on the action potential shape, we observed a comparable amplitude and half-width of the action potentials across the reproductive cycle in PR+ and PR– neurons (Fig. 4*A–E*). The spike amplitude of the posterior neurons of both PR+ and PR– populations is moderately but consistently smaller compared with anterior neurons (Fig. 4*B*,*D*; three-way ANOVA, location *F*_(2,132)_ = 5.74, *p* < 0.01, multiple comparison anterior vs posterior *p* = 0.03) and medial (multiple comparison medial vs posterior *p* < 0.01).

Together, these results suggest that the electrophysiological properties of VMHvl neurons vary across the reproductive cycle, but the observed changes depend on their location in the AP axis. In the sexually receptive phase, anterior PR+ neurons are more hyperpolarized in their resting state and the medial PR+ population exhibits lower excitability.

### The anterior VMHvl presents a higher proportion of rebound firing neurons during the non-sexually receptive phase of the cycle

**Figure 5.**
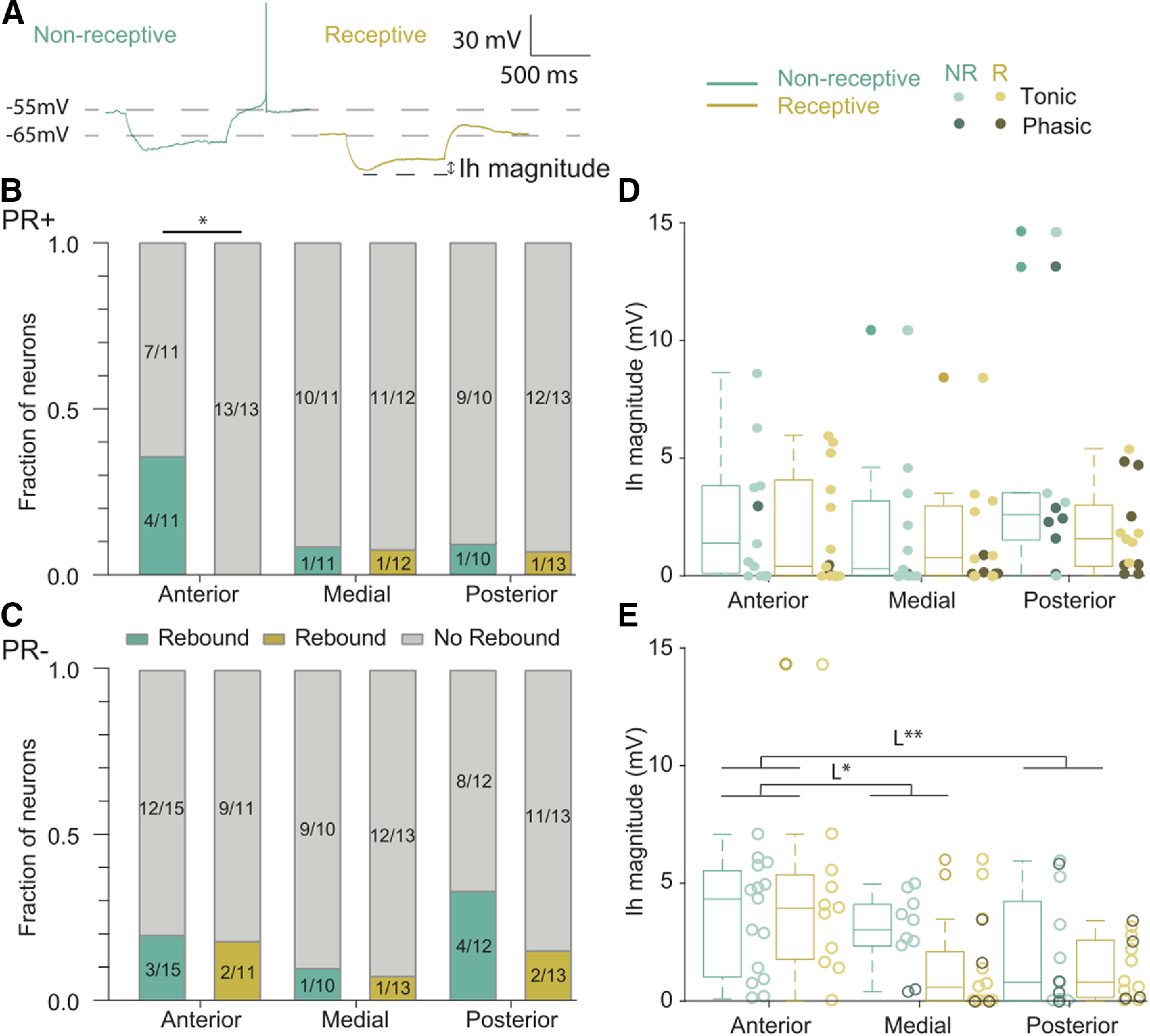
The proportion of anterior PR+ neurons with rebound firing changes across the reproductive cycle. ***A***, Example of recordings of voltage response at –90 pA. Proportion of neurons presenting rebound firing after hyperpolarization of (***B***) PR+ neurons from non-receptive and receptive females, and from (***C***) PR– neurons from non-receptive and receptive females. The anterior PR+ neurons of non-receptive females display a significantly higher fraction of neurons with rebound spikes compared with receptive females (*p* = 0.01) χ^2^ test. ***D***, The Ih amplitude of the PR+ neurons does not change across the reproductive cycle nor the AP axis, while the (***E***) Ih amplitude of PR– neurons varies across the AP axis (three-way ANOVA location/genotype interaction *F*_(2,132)_ = 4.36 *p* = 0.01, two-way ANOVA for PR–, location *F*_(2,68)_ = 6.30, *p* < 0.01, multiple comparison L*, anterior vs medial *p* = 0.04, L**, anterior vs posterior *p* < 0.01). Three-way ANOVA and (***E***) two-way, Sidak correction for multiple comparison; *p < 0.05. Individual data points are shown per group color coded according their tonic or phasic firing type.

Finally, and given that it is well established that hyperpolarizing input can trigger rebound depolarizing responses increasing the firing rate of neurons in a wide variety of brain areas including the hypothalamus (Burdakov et al., 2004; Israel et al., 2008), we sought to characterize the response of VMHvl neurons to hyperpolarizing input. To do so, we quantified the proportion of VMHvl neurons which exhibited rebound firing after hyperpolarization (Fig. 5*A*; –90 pA for 1 s). We observed that 6 out of 32 PR+ neurons of non-receptive females and only two out of 38 PR+ neurons of receptive females fired rebound action potentials (Fig. 5*B*). This difference originates mainly from the anterior PR+ neurons (χ^2^ test, antNon-Rec+ vs antRec+ *p* = 0.01), where four out of 11 neurons of non-receptive females displayed rebound firing while in receptive females none of the 13 recorded neurons produced rebound action potentials. Furthermore, no significant differences were observed between PR+ neurons across the AP axis nor between the PR+ and the PR– populations (Fig. 5*B*,*C*).

Rebound depolarization is caused by the hyperpolarization activated inward current (Ih) which represents a powerful modulator of neuronal firing frequency and timing (Momin et al., 2008). Thus, we quantified the Ih current amplitude in VMHvl neurons (Fig. 5*D*,*E*). A significant difference was obtained in the interaction between location and genotype (three-way ANOVA, location/genotype interaction *F*_(2,132)_ = 4.36 *p* = 0.01), that however, was not large enough to yield statistical differences across groups in the multiple comparisons test. To pinpoint the origin of such changes, we analyzed separately PR+ and PR– populations. Our results show that while the Ih magnitude was unchanged across the AP axis for the PR+ population, the anterior PR– neurons have modestly higher Ih magnitude compared with medial and posterior PR– neurons (two-way ANOVA, location *F*_(2,68)_ = 6.30, *p* < 0.01, multiple comparison anterior vs medial *p* = 0.04, anterior vs posterior *p* < 0.01), however, these differences are not reflected in a significantly different fraction of rebound anterior PR– neurons compared medial and posterior PR– neurons (Fig. 5*C*).

To summarize, the proportion of neurons with rebound firing is fairly homogenous in the VMHvl across the reproductive cycle and independently of genotype, with the exception of its most anterior subdivision, where PR+ neurons exhibit cyclical alterations. Given that these changes are not accompanied by a modulation of Ih current, they likely reflect the change in resting membrane potential that drives receptive neurons away from their firing threshold, causing Ih-mediated depolarization to fail to produce rebound firing (Figs. 3*B*, 5*A*).

### Localized structural plasticity of VMHvl PR+ neurons across the reproductive cycle

**Figure 6.**
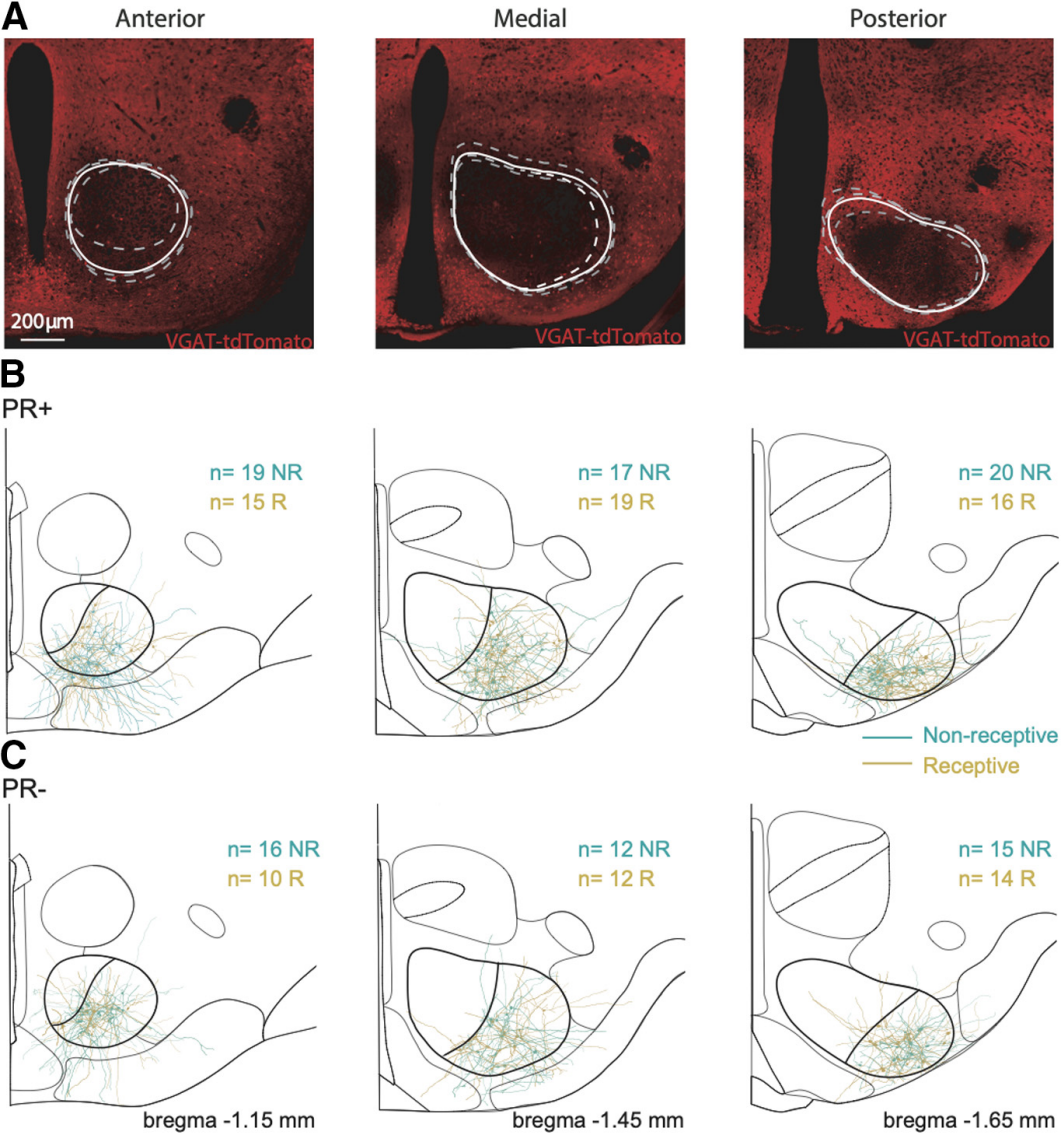
Localization of reconstructed PR+ and PR– neurons across the VMHvl. ***A***, Limits of the VMH obtained with the inspection of GABAergic markers across the AP axis. Schematic representation of the reconstructed PR+ anterior *n* = 19 NR, *n* = 15 R, medial *n* = 17 NR, *n* = 19 R, posterior *n* = 20 NR, *n* = 16 R; (***B***) and PR– anterior *n* = 16 NR, *n* = 10 R, medial *n* = 12 NR, *n* = 12 R, posterior *n* = 15 NR, *n* = 14 R. ***C***, Neurons and their location in the VMHvl and across the AP axis. Scale bar: 200 μm.

To study the impact of the phase of the reproductive cycle on the morphologic properties of VMHvl neurons, we reconstructed and performed morphometric quantifications of the soma area, number of dendrites, primary dendrites and branch points per neuron of PR+ and PR– neurons in different locations across the AP axis of the VMHvl. As previously reported, the VMHvl is primarily composed of glutamatergic neurons, that are surrounded by GABAergic neurons (Yamamoto et al., 2018). In fact, the absence of GABAergic somas has been used to delineate the boundaries of the VMH (Jarvie and Hentges, 2012). Taking advantage of mice expressing the fluorescent reporter tdTomato under the promoter of the vesicular transporter of GABA (VGat-tdTomato; Kaneko et al., 2018) we observed, as expected, lower signal intensity inside the boundaries of the VMH (Fig. 6*A*) that was consistent across the AP regions of the VMH. The limits obtained with the inspection of GABAergic markers were used to delineate the boundaries of the VMHvl, and ensure that the neurons characterized in this study were indeed within the nucleus (Fig. 6*B*,*C*).

The somatic area was unaltered across the reproductive cycle and was not different between the PR+ and PR– populations (Table 2). We observed that PR+ neurons of receptive females exhibit a robust increase in the number of dendrites per neuron across the AP axis (Table 2; three-way ANOVA, genotype/phase interaction *F*_(1,173)_ = 7.85, *p* = 0.01), and a higher number of branching points per neuron compared with neurons originating from non-receptive females (Table 2; three-way ANOVA, genotype/phase interaction *F*_(1,173)_ = 5.81, *p* = 0.02). The changes across the reproductive cycle are specific for the PR+ population, as they were not observed in PR– neurons (Table 2). Both in medial PR+ and PR– neurons, independent of the phase of the cycle, we observed a moderately lower number of primary dendrites per neuron, compared with the anterior and posterior neurons which yielded small yet significant differences across the AP axis (three-way ANOVA, location *F*_(2,173)_ = 3.14, *p* = 0.04).

Neurons in the VMHvl are characterized by having a LPD that can be several fold longer than its SPDs (Calizo and Flanagan-Cato, 2002). Therefore, we analyzed LPD and SPD separately (Table 2). While the length of the LPD of PR+ was unchanged across the reproductive cycle, PR– neurons exhibited longer LPDs in non-receptive females (three-way ANOVA, genotype/phase interaction *F*_(1,168)_ = 6.67, *p* = 0.01). No overall significant differences were found in the SPD length of PR+ and PR– neurons across the cycle and AP axis. Interestingly, the posterior neurons were shown to have shorter LPDs than the anterior and medial neurons regardless of the phase of the cycle or the genotype (three-way ANOVA, location *F*_(2,168)_ = 9.73, *p* < 0.0001). No differences in the length of LPDs and SPDs were found between the PR+ and PR– populations.

**Figure 7.**
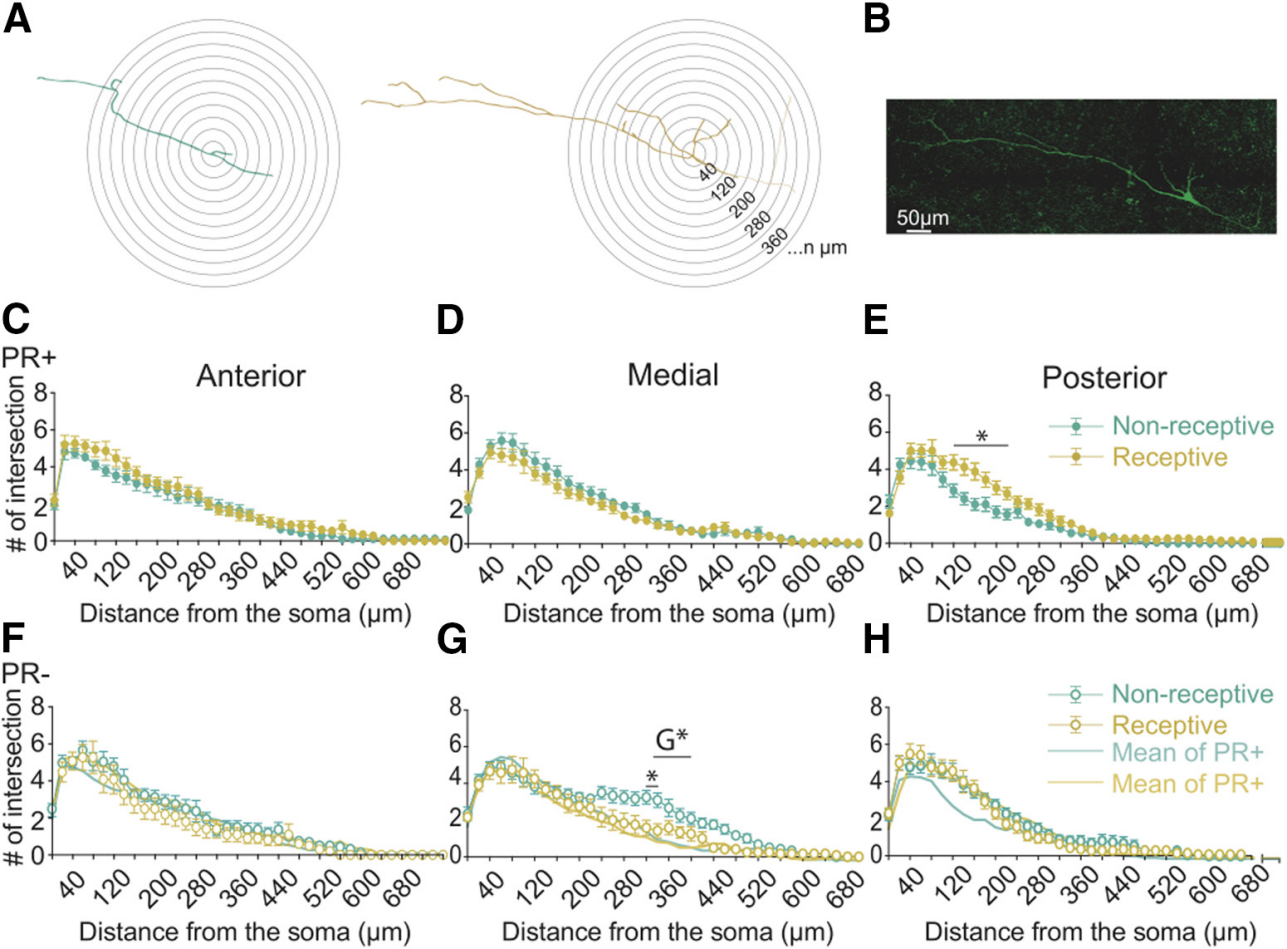
The dendritic properties of PR+ neurons are differently modulated across the reproductive cycle depending on the location in the AP axis and differ from the PR– population. ***A***, Schematic representation of the Sholl analysis of a posterior PR+ neuron of a non-receptive female and a posterior PR+ neuron of a receptive female (the axon, visible in this case, depicted in lighter shade and not considered for analysis). ***B***, Confocal image of the posterior PR+ neuron of a receptive female depicted in A filled with biocytin and stained with streptavidin-Alexa Fluor 488 conjugated. Scale bar: 50 μm. Sholl profiles of (***C–E***) PR+ anterior *n* = 19 NR, *n* = 15 R, medial *n* = 17 NR, *n* = 19 R, posterior *n* = 20 NR, *n* = 16 R and (***F–H***) PR– neurons anterior *n* = 16 NR, *n* = 10 R, medial *n* = 12 NR, *n* = 12 R, posterior *n* = 15 NR, *n* = 14 R across the reproductive cycle. ***E***, The posterior PR+ neurons of receptive females show higher proximal complexity than the ones from non-receptive females (distance from soma × phase of the cycle *F*_(40,136)_ = 3.10, *p* < 0.0001, multiple comparison *p* < 0.001 at 120 μm, *p* < 0.0001 at 140–160 μm, *p* < 0.01 at 180–200 μm, and *p* = 0.04 at 220 μm). ***G***, Medial PR– neurons from receptive females show lower complexity than the ones from non-receptive females (distance from soma × phase of the cycle *F*_(33,726)_ = 2.27, *p* < 0.0001, multiple comparison *p* = 0.03 in 320–340 μm). The medial PR+ neurons have lower distal branching than the medial PR– neurons (distance from soma × genotype *F*_(35,2030)_ = 2.84, *p* < 0.0001, G*, multiple comparison *p* = 0.04 at 320 μm, *p* < 0.01 at 340–360 μm *p* = 0.03 at 380 μm) and (***H***) the posterior PR+ neurons have lower proximal branching than the PR– neurons (distance from soma × genotype *F*_(40,2520)_ = 1.77, *p* < 0.01). Two-way ANOVA with repeated measures. Mean (±SEM). *p<0.05.

In addition to the analysis of dendritic length, we sought to investigate whether the complexity of the dendritic trees was modified across the reproductive cycle. To do so, we used the Sholl method that measures the number of dendritic processes intersections as a function of the radial distance from the soma. No changes were observed in the maximum number of dendritic intersections of PR+ and PR– neurons across the phase of the cycle (Fig. 7*A–H*) nor across their location in the VMHvl, indicating that these neurons reach comparable maximum complexities in their dendritic trees. Nevertheless, the analysis of the Sholl profiles of these neurons revealed that the posterior PR+ neurons of non-receptive females had a significantly lower complexity of their dendritic tree (Fig. 7*E*, repeated measures ANOVA *p* < 0.0001) compared with those from females in the receptive phase. In addition, the medial PR– neurons of non-receptive females had a higher complexity than their counterparts in receptive females (Fig. 7*G*, repeated measures ANOVA *F*_(40,136)_ = 3.10, *p* < 0.0001).

The increased complexity observed in the posterior PR+ neurons of females in the receptive phase was large enough to provide differences when tested with the same method specifically in the proximal branching (Fig. 7*E*; <250 μm from the soma), while the increased complexity of the medial PR– neurons of non-receptive females were observed at more distal parts of the soma (240–340 μm) and were large enough to yield multiple comparisons significant changes (Fig. 7*G*).

We also observed that medial PR+ neurons have lower dendritic branching than medial PR– neurons (Fig. 7*G*; repeated measures ANOVA *F*_(35,2030)_ = 2.84, *p* < 0.0001), particularly in the distal parts from the soma (320–380 μm). The posterior PR+ neurons have lower proximal dendritic branching than the posterior PR– neurons (Fig. 7*H*; repeated measures ANOVA *F*_(40,2520)_ = 1.77, *p* < 0.01). These observations indicate that PR+ neurons have a different dendritic complexity proximal to the cell body compared with that of the PR– population, independent of the phase of the reproductive cycle.

To summarize, similarly to what we report for the electrophysiological properties, the structural properties of PR+ and PR– neurons are diverse, with some varying across the AP axis and the reproductive cycle.

### Dendritic spine density largely differs between the PR+ and PR– populations

**Figure 8.**
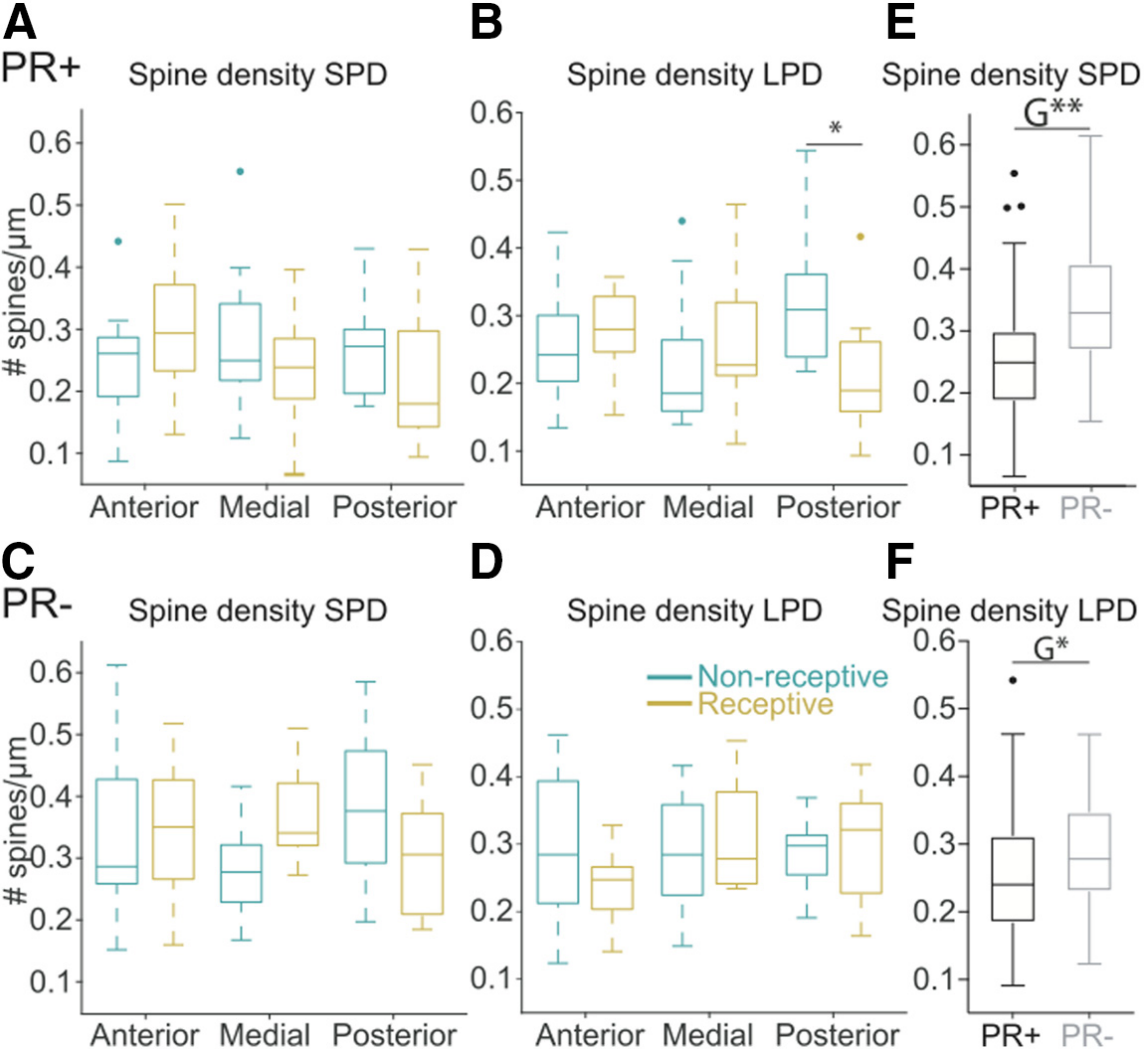
The spine density of the LPD of posterior PR+ neurons changes across the reproductive cycle. ***A***, Spine density of the SPDs anterior *n* = 15 NR, *n* = 13 R, medial *n* = 15 NR, *n* = 17 R, posterior *n* = 15 NR, *n* = 14 R and the (***B***) LPDs anterior *n* = 15 NR, *n* = 13 R, medial *n* = 15 NR, *n* = 17 R, posterior *n* = 13 NR, *n* = 14 R of PR+ neurons across the reproductive cycle. The spine density of the LPDs of posterior PR+ neurons is reduced in receptive females (multiple comparison *p* = 0.0170, causing a change in the three-way interaction between the phase of the reproductive cycle, the location in the AP axis, and the genotype on the LPDs, *F*_(2,150)_ = 4.81, *p* = 0.01). ***C***, Spine density of the SPDs anterior *n* = 15 NR, *n* = 10 R, medial *n* = 12 NR, *n* = 17 R, posterior *n* = 15 NR, *n* = 12 R and the (***D***) LPDs anterior *n* = 15 NR, *n* = 10 R, medial *n* = 12 NR, *n* = 11 R, posterior *n* = 15 NR, *n* = 12 R of PR– neurons across the reproductive cycle. ***E***, PR+ neurons have lower spine density on the SPDs (G**, genotype *F*_(1,153)_ = 26.21, *p* < 0.01) and also (***F***) lower spine density on the LPDs (G*, genotype *F*_(1,150)_ = 7.25, *p* = 0.01) than the PR– neurons. Three-way ANOVA, Sidak correction for multiple comparison. Medians (±25th–75th percentile); *p < 0.05, **p < 0.01

Previous studies have shown that externally primed estrogen exerts effects on the dendritic spine density of VMHvl neurons that are different depending on the type of dendrite, specifically, an increase in spine density on SPDs of VMHvl neurons (Calizo and Flanagan-Cato, 2000) and a decrease of the spine density on LPDs of VMHvl neurons expressing the receptor for estrogen (Calizo and Flanagan-Cato, 2002). In order to determine whether such modulation of the spine density of VMHvl neurons is present in the physiological range of sex hormone fluctuation, the dendritic spine density of PR+ and PR– neurons was analyzed depending not only on their location in the VMHvl, but also on the primary dendrite category across the reproductive cycle of naturally cycling females. Although overall PR+ and PR– neurons have comparable spine densities on SPDs across the reproductive cycle (Fig. 8*A*,*C*) and location, posterior VMHvl neurons in non- receptive females have higher spine density on SPDs compared with posterior VMHvl neurons in the receptive phase resulting in a significant interaction between the phase of the cycle and location within the VMHvl (three-way ANOVA, phase/location interaction *F*_(2,153)_ = 3.88, *p* = 0.02). Furthermore, the PR+ population has significantly lower spine density on the SPDs than PR– neurons (Fig. 8*E*; three-way ANOVA, genotype *F*_(1,153)_ = 26.21, *p* < 0.01).

Similar to what was observed for the spine densities of SPDs, for LPDs we observed that the posterior VMHvl neurons of receptive females present a decreased spine density compared with posterior neurons from females that were non-receptive, which in the case of LPDs the change is specific to PR+ neurons (Fig. 8*B*,*D*; three-way ANOVA, phase/location/genotype interaction *F*_(2,150)_ = 4.81, *p* = 0.01, multiple comparison posNon-Rec+ vs posRec+, *p* = 0.01).

In addition, by comparing PR+ with PR– neurons, we observed that PR+ neurons have lower spine density on the LPDs (Fig. 8*F*; three-way ANOVA, genotype *F*_(1,150)_ = 7.25, *p* = 0.01).

Altogether, we observed that neurons expressing PR have an overall lower spine density when compared with their neighboring PR– neurons. Across the reproductive cycle, posterior PR+ neurons undergo more pronounced structural changes, exhibiting even lower spine density in the receptive phase.

## Discussion

We combined whole-cell recordings with labeling of individual neurons to investigate the structural and electrophysiological properties of PR+ and PR– neurons along the AP axis of the VMHvl and across the reproductive cycle of naturally cycling female mice. First, we report the existence of multiple electrophysiological phenotypes within the VMHvl, including tonic firing neurons (with and without depolarization block) and phasic firing neurons. We show that PR+ cells are distinct from PR– in three major aspects: first, because of structural properties (PR+ neurons have lower spine densities in general); second, because of the great extent to which the properties of PR+ neurons vary across the AP axis; and third because of local changes in the properties of PR+ across the reproductive cycle (which are minimal for the PR– population). Our results further support the existence of subdivisions in the VMHvl and its possible role in coordinating female behavior with the internal reproductive state.

The impact of the reproductive cycle on VMHvl function has been interrogated *in vivo* and *in vitro*, but most studies were performed in females whose ovaries were removed and then supplemented with estrogen and progesterone (Millhouse, 1973; Rubin and Barfield, 1980, 1983a,b; Calizo and Flanagan-Cato, 2000, 2002; Griffin and Flanagan-Cato, 2008; Griffin et al., 2010; for examples of naturally cycling studies, see Nomoto and Lima, 2015; Inoue et al., 2019). While these manipulations are convenient, easing experimental planning and decreasing interindividual variability (with the extra benefit of allowing a reduction in the number of females used), ovariectomized females with hormonal replacement differ substantially from intact females: first, before hormonal replacement the levels of sex hormones are extremely low, as the main source of estrogen and progesterone is absent and second because the hormonal treatment exposes females to concentrations of sex hormones that differ from physiological levels (Liu and Shi, 2015). The expression of hormone receptors, such as the PR, are under the control of sex hormone levels as well (MacLusky and McEwen, 1978), meaning that that the neuronal response is affected at the level of the receiver (receptor) and message (sex hormone). Persistent hormonal replacement of ovariectomized females leads to tumor development (Kordon et al., 1993) further suggesting that the hormonal treatment leads to undesired physiological effects. Finally, the sexual behavior that hormonally treated females exhibits differs from the behavior of naturally cycling females (Zipse et al., 2000), suggesting that the treatment fails to fully recapitulate the effects of natural sex hormones levels. To the best of our knowledge, this is the first study interrogating the intrinsic properties of neurons across the reproductive cycle of naturally cycling females and therefore the results observed reflect endogenous changes. However, we cannot claim that the changes we observe across the reproductive cycle are an effect of sex hormone levels because those were not directly manipulated. Manipulations with more naturalistic levels of sex hormones or local manipulations in the expression levels of sex hormone receptors should be employed to establish a direct causal link between hormonal levels, the structural and physiological properties of VMHvl neurons and the changes observed.

In the present study, we report, for the first time, the existence of different neuronal types in the VMHvl according to their firing pattern. We found tonic and phasic neurons, in both PR+ and PR– populations, intermingled across the AP axis of the VMHvl. However, the presence of phasic neurons was particularly abundant in the posterior VMHvl, where it comprised more than half of the recorded PR+ neurons.

This AP gradient may have strong implications not only for the role of different portions of the VMHvl in controlling behavioral output, but also for the interpretation of activity manipulations, such as the optogenetic control of neural activity. While anterior VMHvl neurons can reach high firing frequencies, posterior phasic neurons present a robust stop of action potential generation after reaching the spiking threshold that does not depend on the current injected in the cell. Importantly, this may be particularly relevant for the interpretation of results where different laser intensities produce diverse behavioral outputs (Lee et al., 2014; Kunwar et al., 2015), as optogenetic activation at particular AP levels may bias the activation of neuronal populations with different intrinsic properties.

Other hypothalamic neuronal populations, such as the tuberoinfundibular dopamine neurons (Lyons et al., 2012) or the gonadotropin-releasing hormone neurons (Jarry et al., 1991) present in the tuberal and preoptic hypothalamus, are known to display phasic firing behavior. Furthermore, the presence of intermingled tonic and phasic firing neuronal population has been previously reported in the magnocellular nucleus of the hypothalamus (Israel et al., 2016), where oxytocin neurons show tonic and vasopressin neurons phasic firing behavior. The differential role of tonic and phasic firing neurons in the control of goal directed behaviors (Grace et al., 2007; Budygin et al., 2020) but also in their contributions for encoding stimulus-environment features (Pilkiw et al., 2017) has been widely studied throughout the brain. In addition, it is known that changes in voltage-sensitive channels (Suzuki and Rogawskit, 1989), that can be triggered by neuromodulation (Lyons et al., 2012; Valentino and Van Bockstaele, 2015), are able to mediate the dynamic transition between tonic to phasic firing regimes. In the VMHvl, whether tonic and phasic firing neuronal populations, or the dynamic transition between these firing regimes, contribute to different aspects of socio-sexual behavior remains elusive and should be the focus of future studies.

Our results indicate that PR+ cells are distinct from their neighbors, a difference that is probably established early in development, and later on by the fact that their intrinsic properties of this population can be directly affected by estrogen and progesterone, as PR+ neurons co-express ER (Blaustein and Turcotte, 1985; Hashikawa et al., 2017; Sá and Fonseca, 2017). Interestingly, the properties of PR– neurons were similar along the AP axis and across the reproductive cycle, with the exception of changes in the dendritic complexity of neurons in the medial VMHvl. This observation may be explained by the fact that not all ER+ neurons co-express PR. ER+/PR– neurons were not labeled with our genetic strategy, but their properties are sensitive to fluctuating levels of estrogen (Calizo and Flanagan-Cato, 2000, 2002). Intersectional strategies are needed to address this question.

Interestingly, although anterior PR+ neurons had a comparable excitability profile across the reproductive cycle, the resting potential of neurons in the receptive phase was significantly hyperpolarized. This change in resting potential likely underlies a moderate increase in the threshold to fire in these neurons, which required more current to start generating action potentials. Even if this trend did not reach significant differences with our current statistical power, the visual difference between anterior non-receptive and receptive PR+ neurons can hardly be overlooked (Fig. 3*C*). In addition, the anterior PR+ neurons of receptive females do not show hyperpolarized-induced rebound firing. The fact that these neurons have a more hyperpolarized resting potential, thus more distant from the firing threshold, might explain the fact that the same magnitude of Ih current fails to evoke rebound firing in anterior PR+ at the receptive phase (Fig. 5*B*). We also report a robust increase of the firing rate under same current injections in medial VMHvl neurons obtained from females in the non-receptive phase compared with those in the receptive phase. These changes were not accompanied by changes in capacitance or Rm across the reproductive cycle of medial neurons, thus it is unlikely that the changes in firing rate are caused by differences in the passive propagation of current into the neuron and instead points toward a modulation of voltage-sensitive channels in the medial PR+ neurons, that may be mediated by progesterone (Scharfman and MacLusky, 2006). Overall, these results suggest that the anterior and medial VMHvl have the potential to generate more action potentials in non-receptive females, which is in seeming disagreement with the enhanced-male evoked responses that we previously observed, at the population level, in the VMHvl of sexually receptive female mice (Nomoto and Lima, 2015). However, the results of this study were obtained with extracellular recordings of non-identified neurons and therefore we do not know whether they were PR+ or PR–. Also, in the present study, we investigated the intrinsic properties of VMHvl neurons and cannot make any claim regarding the driving input they receive (synaptic and/or neuromodulatory). The VMHvl receives indirect input from the vomeronasal organ, which includes some neurons whose activity is increased in response to male stimuli when females are sexually receptive (Dey et al., 2015). It is conceivable that the enhanced VMHvl activity that we previously observed reflects modifications at the sensory level.

It has been previously reported that hormonal treatment in ovariectomized rats reduced the amount and length of secondary dendrites of a non-defined VMHvl neuronal population (Griffin and Flanagan-Cato, 2009). Even if in seeming contradiction with our findings, since we report the opposite effect in dendritic complexity and unchanged dendritic lengths, we would like to point out several possible reasons for such difference. First, the changes we observed are specific to the posterior PR+ population (and absent in our sample of PR– neurons), therefore, sampling VMHvl neurons independently of their genotype could have masked a specific plasticity process in posterior PR+ neurons. Second, in our dataset we observed that medial PR– neurons undergo a reduction in dendritic complexity that goes in line with previous reports. Thus, pooling samples from different levels of the VMHvl may mask changes, or alternatively sampling exclusively in a single AP region of the VMHvl could lead to findings that are limited to that level alone, and thus not generalizable to the whole VMHvl. Interestingly, posterior PR+ neurons did not only undergo changes at the level of their dendritic complexity, but also at the level of spine density, which was reduced in the receptive phase specifically in their LPDs. The fact that such dendritic spine changes are specific to LPDs may suggest that some pathway specific plasticity is happening during the reproductive cycle. Although it has been previously hypothesized that SPDs may preferentially integrate local excitatory inputs, while LPDs may preferentially interact with long range inputs, inhibitory neurons of the shell and neuropeptides (Griffin and Flanagan-Cato, 2011; Yamamoto et al., 2018), as of yet, no circuit mapping study has quantitatively explored what is the anatomic origin of the inputs. Thus, both the anatomic origin of those inputs and their possible relevance for the dendritic coding presented here needs to be further investigated. Our findings suggest a modification of the overall synaptic weights that the LPDs and SPDs will have in the dendritic integration for output generation, with a bias toward the inputs contacting SPDs.

It is worth noting that while the spine densities we observed are comparable to the ones reported in previous studies (Calizo and Flanagan-Cato, 2002), the dendritic lengths we report both for PR+ and for PR– neurons are several fold longer than those previously reported (Calizo and Flanagan-Cato, 2002). These changes may be explained by the differences in the technical approaches used to investigate neuronal structure. While previous reports have used fixed histologic slices (100–150 μm) filled a posteriori with lucifer yellow, in the present study we have used acute slices for *ex vivo* electrophysiological recordings (300 μm), in which neurons were filled with biocytin during their electrophysiological monitoring. Thus, several key differences may explain the contradictory results: first, the thickness of the slice, which could allow us to track dendrites for longer extensions; second, the fact that neurons in acute slices for *ex vivo* recordings reseal their membranes, producing a visible thickening in the extremity of their cut dendrites, thus allowing us to recognize incomplete dendrites with our technical approach.

In summary, we have observed multiple electrophysiological phenotypes within the VMHvl and a surprising diversity of structural and physiological plasticity processes both along the AP axis of the VMHvl, and across the reproductive cycle, within the genetically defined population of PR+ cells. These findings highlight the repertoire of local plasticity rules across the VMHvl that is probably explained by the different specialized transcriptomic clusters previously characterized across the AP extension of the VMHvl (McClellan et al., 2006; Kim et al., 2019), supporting the existence of subdivisions in the VMHvl and a more complex role of this hypothalamic structure in socio-sexual behavior, which has been recently acknowledged (Yang et al., 2013; Sakurai et al., 2016; Hashikawa et al., 2017; Inoue et al., 2019; Wang et al., 2019). The existence of multiple subdivisions that undergo local changes in response to sex hormone levels hint to the hypothesis that this hypothalamic structure might be able to control a complex and coordinated set of responses in reaction to a ubiquitous message, the systemic levels of sex hormones in circulation, from metabolism to sexual receptivity. This idea is supported by the connectivity of ER+ neurons, which present a high degree of output divergence, with anterior ER+ neurons projecting to premotor areas, while posterior ER+ are engaged in amygdalo-hypothalamic loops (Lo et al., 2019). Similar circuit motifs have been described in other hypothalamic nuclei, such as the organum vasculosum of the lamina terminalis (OVLT), where different subnuclei can coordinate a multipronged response in reaction to thirst (Graebner et al., 2015). Altogether, it becomes evident that acknowledging the different neuronal properties within the AP axis of the VMHvl is crucial to have a holistic understanding of its involvement in behavior and further studies with focal manipulations are needed to understand the functional role of observed local properties and their modulation by the reproductive cycle.

## Synthesis

Reviewing Editor: Michael Michaelides, NIDA-NIH Decisions are customarily a result of the Reviewing Editor and the peer reviewers coming together and discussing their recommendations until a consensus is reached. When revisions are invited, a fact-based synthesis statement explaining their decision and outlining what is needed to prepare a revision will be listed below. The following reviewer(s) agreed to reveal their identity: NONE. Note: If this manuscript was transferred from JNeurosci and a decision was made to accept the manuscript without peer review, a brief statement to this effect will instead be what is listed below.

Reviewer 1

Manuscript Dias et al. eN-TNWR-0049-21X

This is a very interesting study on Progesterone expressing and non expressing cells in the VMHvl which explores some electrophysiological and anatomical properties along the >Anterior posterior Axis and in receptive and non-receptive females, finding that subpopulation of PR+ neurons present characteristics that depend on their anatomical location and the reproductive stage. The study is well conducted and the manuscript is well-written. However, some of the results described are difficult to interpret form the current form in which data is present, i.e some statistical comparisons are not clear in the figures. Some homogenization is needed in Figure´s legends in terms of the information contained there, and some points need further clarification. Please, see below.

1) The oestrous cycle was assessed in the morning, which in the inverted light cycle corresponds to the dark period. At what time were the animals sacrificed? Meaning, were the animals sacrificed during the dark phase (in a regular light cycle, the “afternoon”) of proestrus? In the transition from proestrus to oestrous? Were animals in oestrous and proestrus pooled under the same category? Since animals at the start of proestrus, mice are still non-receptive, it would be important to clarify this point.

2) Alexa 488 used to recognize patched cells and EYFP, which is expressed in PR-Cre-EYFP animal have very similar excitation/emission spectrum. How was the microscopy performed to avoid overlap of both signals? Which microscope and software were used for the analysis? (Is it the same as for 45 micrometres coronal sections?). That is not clear. Can the authors clarify how was the procedure to classify cells in PR positive or negative? Was it done during the recording and then confirmed after with the biocytin-Alexa 488 streptoavidin staining?

3) On page 14 it is explained that the slices containing biocytin filled cells were immunostained for reconstitution of neuronal structure. What does immunostained refer to? To Streptoavidin-Alexa 488 staining?

4) Statistics: It is not clear in the figure legends which data sets were Kruskal-Wallis tests used. Also, the figure´s legends sometimes state the statistical test use and in some this information is absent. Could you unify the legends and for every significance or set of figures clarify in the legend the test used?

5) Fig. 1. Figure Legend refers to Letters G, I and H but those are missing in Figure 1. Please complete the Figure.

6) Can the authors show statistically significant in Table 1 or Table 1 legend?

7) In Figure 2 A, the representative trace show a remarkably decreased frequency in PR+ neurons from receptive respect to non-receptive females, in response to +30 or +60 pA pulses. As for the figure´s title, it is understood that correspond to Anterior cells. However, in Fig. 1 with an equivalent pulse, no differences were observed in the frequency between responsive and non-responsive females for the Anterior area. Can you please clarify this discrepancy or if the traces correspond to posterior cells? Please add this information to the Figure legend.

8) It would be helpful to understand the results that the differences found in Latency to a spike in posterior neurons were noted in the graph (in Fig. 2F and H (as in the Results section))

9) In Fig 3 it would make the figure clearer if the step protocol used is represented in the figure or clarified in the Figure legend.

10) In Fig. 3 D, the legend states: “The spike amplitude of PR- neurons in maintained across the cycle, however, it differs across the AP axis (p<0,01)” However, no significance is noted in the graph. Those differences described, to which are do they correspond to?

11) In Fig.3 A the action potential illustrated for Non-receptive and receptive females show a marked difference in the HW, but the analysis of this parameter shows no change. Can the authors show a trace that better represents the data?

12) Can the authors make clear the n in the figure legends (except for those including pie charts, in which the n is clear)?

13) It is clear the exemplification of the firing patterns in Fig. 4A-C. But how were the neurons classified after recording in tonic or phasic, meaning, what numeric/objective criteria were used to classify cells as tonic or phasic?

14) In Fig. 4. The difference in the posterior PR+ neurons of p<0,001, to what comparison does it correspond to? In both Fig. 4 and 5, a table summarizing the statistical comparisons among the groups will be useful to understand better which comparisons were made. Fig. 4: Aren´t there statistical significant changes in the tonic with depolarization block group in receptive and non-receptive females? Would it be relevant to compare PR+ vs PR- ?

15) Fig. 5 E: The significant difference in the Ih amplitude in PR neurons is not noted in the graph. Which area(s) is (are) different form which is then not clear.

16) Fig. 6: It would make the figure clearer if it is referenced what does the red represent (although in the text, the information in the figure is valuable). Is the proportion of PR+ vs PR- cells constant across the AP axis?

17) Table 2: Can the authors add to the table significant differences among the groups for the parameters evaluated?

18) Fig.7: B, E, Fig 8: The fact that the data of PR+ and PR- cells are in different graphs makes the result a little unclear. It would be helpful to see the numbers and the comparisons made between PR+ and PR- cells in an additional graph or table. Can the authors illustrate a Sholl analysis of a cell in receptive and non-receptive female showing the difference in the dendrites? Can the authors add a picture of a filled cell to illustrate a PR+ neuron?

19) Discussion: Page 27: Line 4: “Our results further support the existence of subdivisions in the VMHvl and its role in coordinating female behaviour with the internal reproductive state”. Since the study does not include a functional evaluation of the proposed subpopulations, the results support the existence of such a subdivision, but not its functional role.

20) As explained in the discussion, PR expression may vary along the oestrous cycle. But in the model used (PR-Cre-EYFP), such variations won´t be evident.

Minor comments

21) Use the subscript form for correct formula writing in the Materials and Methods section.

22) Were the brains frozen before microtome cutting?

23) What is it ImageJ Sofware179?

Reviewer 2

Dias & Gutierrez-Castellanos et al. aim to characterize the electrophysiological properties and plasticity of VMHvlPR neurons at two different time points of the estrous cycle when female mice are either receptive or non-receptive. This study builds upon the original observations of Inoue et al., 2019 and Stagkourakis et al., 2020, which describe unprecedented levels of plasticity in VMHvl. Using the two conditions of female receptivity as the testbed for their experiments, they investigate the electrophysiological characteristics of VMHvlPR+ and VMHvlPR- cells, such as the I-V curve, resting membrane potential, action potential features, among others. The analytical approaches used by the authors are good.

A meticulous, careful investigation of what the authors set out to do will be undoubtedly useful and informative for the field. However, I do have some comments for revising the manuscript.

Major points

1. Unpublished data from other labs suggest that the male VMHvl “houses” four distinct electrophysiological phenotypes (sometimes intermixed), specifically:

a) regular tonic firing cells, which respond with regular tonic firing to a depolarizing square pulse

b) late-firing neurons (with an IA-like conductance), which respond with a delayed high-interval AP firing to a depolarizing square pulse

c) bursty cells which exhibit fast firing and fast induction of depolarization block following delivery of a depolarizing square pulse, and

d) cells that express Ih (and/or T-type Ca2+ channels), featuring rebound depolarization following delivery of a hyperpolarizing square pulse.

The authors have indeed identified these distinct electrophysiological phenotypes in the female VMHvl, but they only present them “late” in the manuscript, in Figure 4.

1a. Since this finding is not published in the literature, it should be the first thing the authors illustrate in their manuscript, and move these findings to Figure 1. It is important to make it clear to the reader that there is not just a single electrophysiological phenotype in VMHvl. In addition, the authors should include the IV curves and raw traces of the three distinct electrophysiological phenotypes they describe.

1b. However this point (multiple electrophysiological phenotypes), complicates the interpretation of the findings illustrated in Figure 1, Table 1, Figure 2 and Figure 3. Once this necessary information is presented, the reader will wonder whether the comparisons currently presented in Figures 1-3 should take into consideration the electrophysiological phenotype. For example, it seems inappropriate to compare the input resistance of tonic firing cells from receptive mice with phasic firing cells from non-receptive mice, and suggest that there is a difference. I would therefore recommend that the authors re-analyze the comparisons they have illustrated in Figure 1, Table 1, Figure 2 and Figure 3, by clustering groups according to electrophysiological phenotype.

2. In Figure 5B the authors illustrate that in receptive mice, there are no rebound cells in the anterior VMHvl. When looking however at the quantification of the Ih magnitude (Figure 5D) in the anterior VMHvl, I was expecting the box plot to be at 0, given that there are no cells with rebound. Why is this not the case?
